# Supplementary material for: Overcoming optical losses in thin metal-based recombination layers for efficient n-i-p perovskite-organic tandem solar cells
Source: Nat Commun. 2025 Jan 2;16:154. doi: 10.1038/s41467-024-55376-7 (PMC11696673; doi:10.1038/s41467-024-55376-7)
Supplement: Supplementary file 1 — Supplementary Information [file 41467_2024_55376_MOESM1_ESM.pdf]

Supplementary Information

Overcoming Optical Losses in Thin Metal-Based Recombination Layers for Efficient n-i-p Perovskite-Organic Tandem Solar Cells

Jingjing Tian,[1,2] Chao Liu,[1,3,*] Karen Forberich,[3] Anastasia Barabash,[1] Zhiqiang Xie,[1] Shudi Qiu,[1] Jiwon Byun,[4] Zijian Peng,[1,2] Kaicheng Zhang,[1] Tian Du,[3] Sanjayan Sathasivam,[5] Thomas J. Macdonald,[6] Lirong Dong,[1,2] Chaohui Li,[1,2] Jiyun Zhang,[3] Marcus Halik,[4] Vincent M. Le Corre,[3] Andres Osvet,[1] Thomas Heumüller,[1,3] Ning Li,[7] Yinhua Zhou,[8] Larry Lüer,[1,*] Christoph J. Brabec[1,3,*]

[1]Institute of Materials for Electronics and Energy Technology (i-MEET), Department of Materials Science and Engineering, Friedrich-Alexander-Universität Erlangen-Nürnberg, Martensstraße 7, 91058 Erlangen, Germany

[2]Erlangen Graduate School in Advanced Optical Technologies (SAOT), Paul-Gordan-Straße 6, 91052 Erlangen, Germany

[3]Helmholtz-Institute Erlangen-Nürnberg for Renewable Energy (HI ERN), Immerwahrstraße 2, 91058 Erlangen, Germany

[4]Organic Materials & Devices, Institute of Polymer Materials, Friedrich-Alexander-Universität Erlangen-Nürnberg, Interdisciplinary Center for Nanostructured Films (IZNF), Cauerstraße 3, D91058 Erlangen, Germany

[5]School of Engineering, London South Bank University, London, SE1 0AA, UK

[6]Department of Electronic & Electrical Engineering, University College London, London WC1E 7JE, U.K.

[7]Institute of Polymer Optoelectronic Materials & Devices, Guangdong Basic Research Center of Excellence for Energy & Information Polymer Materials, State Key Laboratory of Luminescent Materials & Devices, South China University of Technology, Guangzhou 510640, P. R. China

[8]Wuhan National Laboratory for Optoelectronics, Huazhong University of Science and Technology, 430074 Wuhan, China

*Correspondence:
c.liu@fz-juelich.de (C.L.),
larry.lueer@fau.de (L.L.),
christoph.brabec@fau.de (C.B.)

This PDF file includes:

Notes S1-2

Figures S1-S45

Tables S1-S11

Supplementary References.

Note S1: Surface free energy

The surface free energy of solids (γ_s) can be modeled according to the assumption that the total surface energy of solids is contributed from polarity and dispersity ($\gamma_s = \gamma_s^p + \gamma_s^d$).[1-3]

Basically, the liquid droplet on flat surface following Young's equation:

$$\gamma_{sv} - \gamma_{sl} = \gamma_{lv} cos\theta$$

where γ_{sv}, γ_{sl} and γ_{lv} refer to interfacial tensions at solid-vapor, solid-liquid and liquid-vapor interfaces, respectively. θ is the contact angle of a liquid on a solid surface.

By combining Good's equation:

$$\gamma_{sl} = \gamma_s + \gamma_l - 2\left(\gamma_s^d \gamma_l^d\right)^{1/2} - 2\left(\gamma_s^p \gamma_l^p\right)^{1/2}$$

the Owens-Wendt equation is as following:

$$\frac{\gamma_l(1 + \cos\theta)}{2(\gamma_l^d)^{1/2}} = (\gamma_s^p)^{1/2} \frac{(\gamma_l^p)^{1/2}}{(\gamma_l^d)^{1/2}} + (\gamma_s^d)^{1/2}$$

Therefore, by plotting $(\gamma_l^p)^{1/2}/(\gamma_l^d)^{1/2}$ versus left part of above equation, the surface free energy of a solid ($\gamma_s = \gamma_s^p + \gamma_s^d$) can be calculated.

The above Owens-Wendt model is based on a smooth and homogeneous surface. Furthermore, according to Wenzel Theory, the roughness can be specified by correcting θ with the roughness factor (*r*):

$$Cos\,\theta_W = r\cos\theta_Y$$

$$r = \frac{A^{actual}}{A^{projected}}$$

Where θ_W is the predicted Wenzel contact angle, θ_Y is the Young contact angle, A^{actual} is the solid-liquid actual surface area, $A^{projected}$ is the projected area.

Note S2: Haze transmittance

The haze transmittance, which is often used to estimate the light-scattering property of the samples, is determined by the following equation:

$$\text{Haze} = \left(\frac{T_d}{T_t} - \frac{T_i}{T_b}\right) * 100\%$$

where T_d and T_t represent the diffused and total transmission spectra of given samples, whereas T_i belongs to the light passthrough the integrating sphere undisturbed and T_b exits from an integrating sphere closed with a white standard (i.e., baseline of the instrument).

Table S1. Device architectures and performance of well-performing P-O-TSCs.

	Device Structure	E_g (eV)	V_{OC} (V)	J_{SC} (mA/cm^2)	FF (%)	PCE (%)	Ref.
p-i-n PSC	ITO/NiO_x/$FA_{0.8}MA_{0.02}Cs_{0.18}PbI_{1.8}Br_{1.2}$/$C_{60}$/BCP/Ag	1.77	1.103	16.1	83.16	14.5	4
p-i-n OSC	ITO/MoO_x/PBDBT-2F:Y6:$PC_{71}BM$/TPBi/Ag	1.41	0.842	25.0	77.3	16.3	(2020)
p-i-n TSC	ITO/NiO_x/PVK/C_{60}/BCP/Ag/MoO_x/BHJ/TPBi/Ag		1.902	13.05	83.1	20.6	
p-i-n PSC	ITO/poly-TPD/$MA_{0.96}FA_{0.1}PbI_2Br(SCN)_{0.12}$/PCBM/BCP/Ag	1.70	1.19	18.7	78.4	17.4	5
p-i-n OSC	ITO/MoO_3/PM6:CH1007/PFN-Br/Ag	1.35	0.83	26.9	71.4	15.9	(2022)
p-i-n TSC	ITO/poly-TPD/PVK/PCBM/BCP/Au/MoO_3/BHJ/PFN-Br/Ag		1.96	13.8	78.4	21.2	
p-i-n PSC	ITO/NiO_x/BPA/$Cs_{0.25}FA_{0.75}Pb(I_{0.6}Br_{0.4})_3$/$C_{60}$/BCP/Ag	1.79	1.26	17.9	78.9	17.8	6
p-i-n OSC	IZO/MoO_x/PM6:Y6/PBDIT-F3N/Ag (sputter)	1.36	0.845	26.8	74.8	16.75	(2022)
p-i-n TSC	ITO/NiO_x/BPA/PVK/C_{60}/BCP/IZO/MoO_x/BHJ/PBDIT-F3N/Ag		2.06	14.83	77.2	23.60	
p-i-n PSC	ITO/PTAA/Meo-2PACz/$FA_{0.8}Cs_{0.2}Pb(I_{0.5}Br_{0.5})_3$/PCBM/AZO-NP/ALD-$SnO_x$/Ag	1.85	1.34	15.6	81	16.8	7
p-i-n OSC	ITO/MoO_x/PM6:Y6/C_{60}/BCP/Ag	1.33	0.87	26.7	75	17.5	(2022)
p-i-n TSC	ITO/PTAA/Meo-2PACz/PVK/PCBM/AZO-NP/ALD-SnO_x/ALD-InO_x/ MoO_x/BHJ/C_{60}/BCP/Ag		2.15	14.0	80	24.0	
p-i-n PSC	ITO/2PACz/$FA_{0.6}MA_{0.4}Pb(I_{0.6}Br_{0.4})_3$/$C_{60}$/BCP/Ag	1.79	1.25	16.9	83.0	17.6	8
p-i-n OSC	ITO/MoO_x/PTB7-Th:BTPV-4Cl-eC9/PDINN/Ag	1.20	0.65	28.6	69.2	12.6	(2022)
p-i-n TSC	ITO/2PACz/PVK/C_{60}/BCP/Ag/MoO_x/BHJ/PDINN/Ag		1.88	15.7	74.6	22.0	
p-i-n PSC	ITO/PVBT-SO_3/$MAPbI_{2.95}Cl_{0.05}$/$PC_{61}BM$/C_{60}-ionene/Ag	1.6	1.08	16.2	80	14.0	9
p-i-n OSC	ITO/MoO_3/PM6:Y6/C_{60}-ionene/Ag	1.33	0.86	25.2	72	15.6	(2022)
p-i-n TSC	ITO/PVBT-SO_3/PVK/$PC_{61}BM$//C_{60}-ionene/Ag/MoO_3/BHJ/C_{60}-ionene/Ag		1.92	12.6	79	19.2	
p-i-n PSC	ITO/poly-TPD/$MA_{1.06}PbI_2Br(SCN)_{0.12}$/PCBM/BCP/Ag	1.70	1.16	17.3	76.3	15.3	10
p-i-n OSC	ITO/MoO_3/PM6:Y6/PFN-Br/Ag	1.33	0.815	26.4	74.6	15.8	(2022)
p-i-n TSC	ITO/poly-TPD/PVK/PCBM/BCP/Au/MoO_3/BHJ/PFN-Br/Ag		1.94	13.12	78.7	20.03	
p-i-n PSC	MgF_2/ITO/2PACz/$FA_{0.8}Cs_{0.2}Pb(Br_{0.4}I_{0.6})_3$/$C_{60}$/BCP/Ag	1.78	1.18	18.1	67.1	14.3	11
p-i-n OSC	MgF_2/ITO/PEDOT:PSS/PTB7-Th:IEICO-4F/PFN-Br/Ag	1.24	0.71	24.9	32.8	11.1	(2022)
p-i-n TSC	MgF_2/ITO/2PACz/PVK/C_{60}/BCP/SnO_2/PEDOT:PSS/BHJ/PFN-Br/Ag		1.85	13.8	70.5	18.0	
p-i-n PSC	ITO/DC-PA/$Cs_{0.2}FA_{0.8}Pb(I_{0.6}Br_{0.4})_3$/PI/$C_{60}$/BCP/Ag	1.81	1.351	17.52	82.74	19.58	12
p-i-n OSC	ITO/MoO_x/PM6:Y6:PC71BM/PNDIT-F3N/Ag	1.33	0.840	26.58	74.43	16.62	(2024)
p-i-n TSC	ITO/DC-PA/PVK/PI/C_{60}/BCP/Au/MoO_x/BHJ/PNDIT-F3N/Ag		2.151	14.36	81.65	25.22	

p-i-n PSC	ITO/NiO_x/2PACz/$FA_{0.8}Cs_{0.2}PbI_{1.6}Br_{1.4}$-$Pb(SCN)_2$/PEAI/$C_{60}$/BCP/Ag	1.84	1.32	17.06	84.21	18.96	13
p-i-n OSC	ITO/MoO_x/2PACz/D18-Cl:N3:$PC_{61}BM$/C_{60}/BCP /Ag	1.38	0.856	28.08	78.41	18.85	(2024)
p-i-n TSC	ITO/NiO_x/2PACz/PVK/C_{60}/BCP/Ag/MoO_x/2PACz/BHJ/C_{60}/BCP/Ag		2.12	14.68	82.97	25.82	
p-i-n PSC	ITO/NiO_x/Me-4PACz/$Cs_{0.25}FA_{0.75}Pb(I_{0.5}Br_{0.5})_3$/PCBM/BCP/Ag	1.86	1.366	16.10	84.20	18.52	14
p-i-n OSC	ITO/MoO_x/PM6:BTP-eC9/PDINN/Ag	1.39	0.852	26.15	74.89	16.68	(2024)
p-i-n TSC	ITO/NiO_x/Me-4PACz/PVK/PCBM/AZO/ITO/MoO_x/BHJ/PDINN/Ag		2.144	14.65	80.02	25.13	
n-i-p PSC	ITO/ZnO/$CsPbI_2Br$/P3HT/MoO_x/Ag	1.85	1.15	13.48	78.0	12.64	15
n-i-p OSC	ITO/ZnO/PTB7-Th:IEICO-4F/MoO_x/Ag	1.25	0.70	24.07	66.0	11.02	(2020)
n-i-p TSC	ITO/ZnO/PVK/P3HT/MoO_x/Au/ZnO/BHJ/MoO_x/Ag		1.73	12.94	80.1	18.04	
n-i-p PSC	ITO/ZnO/SnO_2/$CsPbI_2Br$/PDCBT/MoO_3/Ag	1.90	1.269	13.48	84.79	14.51	16
n-i-p OSC	ITO/ZnO/PM6:Y6/MoO_3/Ag	1.33	0.78	25.69	71.83	14.42	(2020)
n-i-p TSC	ITO/ZnO/SnO_2/PVK/PDCBT/MoO_3/Ag/ZnO/BHJ/MoO_3/Ag		1.95	12.46	75.59	18.38	
n-i-p PSC	ITO/ZnO/$CsPbI_2Br$/polyTPD/MoO_3/Ag	1.92	1.19	15.2	79.2	14.5	17
n-i-p OSC	ITO/ZnO/PM6:Y6-BO/MoO_3/Ag	1.30	0.826	26.2	74.4	16.1	(2021)
n-i-p TSC	ITO/ZnO/PVK/polyTPD/MoO_3/Ag/PFN-Br/BHJ/MoO_3/Ag		1.96	13.30	80.3	20.9	
n-i-p PSC	ITO/SnO_2/$CsPbI_{1.8}Br_{1.2}$/TACl/PBDB-T/MoO_3/Ag	1.93	1.25	14.34	79.39	14.32	18
n-i-p OSC	ITO/ZnO/PFN/PM6:Y6/MoO_3/Ag	1.40	0.83	26.25	73.8	16.08	(2021)
n-i-p TSC	ITO/SnO_2/PVK/TACl/PBDB-T/MoO_3/Au/ZnO/PFN/BHJ/MoO_3/Al		2.05	13.36	76.82	21.04	
n-i-p PSC	ITO/SnO_2/ZnO/$CsPbI_2Br$/PTAA/MoO_3/Ag	1.90	1.23	15.03	78.2	14.48	19
n-i-p OSC	ITO/ZnO NPs/D18:Y6/MoO_3/Ag	1.33	0.85	24.22	73.7	15.23	(2021)
n-i-p TSC	ITO/SnO_2/ZnO/PVK/PTAA/MoO_3/Au/ZnO NPs/BHJ/MoO_3/Ag		2.05	13.07	75.3	20.18	
n-i-p PSC	ITO/ZnO/SnO_2/$CsPbI_2Br$/PTAA/MoO_3/Ag	1.90	1.271	14.79	78.1	14.7	20
n-i-p OSC	ITO/PFN-Br/PM6:Y6/MoO_3/Ag	1.33	0.846	22.37	70.7	13.4	(2022)
n-i-p TSC	ITO/ZnO/SnO_2/PVK/MoO_3/Ag/PFN-Br/BHJ/MoO_3/Ag		2.097	13.09	75.1	20.6	
n-i-p PSC	ITO/ZnO/SnO_2/MAFm/$CsPbI_2Br$/MAFm/PDCBT/MoO_3/Ag	1.90	1.347	15.10	83.7	17.00	21
n-i-p OSC	ITO/ZnO/BCP/PM6:CH1007/MoO_3/Ag	~1.3	0.803	26.36	71.82	15.21	(2023)
n-i-p TSC	ITO/ZnO/SnO_2/MAFm/PVK/MAFm/PDCBT/MoO_3/Au/ZnO/BCP/ BHJ/MoO_3/Ag		2.10	14.23	77.70	23.21	
n-i-p PSC	ITO/ZnO/SnO_2/$CsPbI_{1.9}Br_{1.1}$/D18-Cl/MoO_3/Al	1.91	1.36	15.14	81.43	16.77	22
n-i-p OSC	ITO/ZnO/PFN/D18-Cl:N_3:$PC_{61}BM$/MoO_3/Al	1.38	0.844	26.60	76.94	17.27	(2023)
n-i-p TSC	ITO/ZnO/SnO_2/PVK/D18-Cl/MoO_3/Au/ZnO/PFN/BHJ/MoO_3/Al		2.15	13.43	80.25	23.17	

n-i-p PSC	ITO/Cl@MZO/$CsPbI_2Br$/PM6/MoO_x/Ag	1.91	1.305	15.54	83.98	17.05	23
n-i-p OSC	ITO/PFN-Br/PM6:BTP-eC9/MoO_x/Ag	1.39	0.833	26.32	73.65	16.15	(2023)
n-i-p TSC	ITO/Cl@MZO/PVK/PM6/MoO_x/Ag/PFN-Br/BHJ/MoO_x/Ag		2.152	13.89	80.57	24.07	
n-i-p PSC	ITO/ZnO/SnO_2/$CsPbI_2Br$ (~285 nm)/D18-Cl/MoO_x/Ag	1.89	1.40	13.84	82.54	15.98	
n-i-p OSC	ITO/ZnO/D18-Cl:L8-BO/2PACz-Cl/MoO_x/Ag	1.46	0.937	24.07	74.80	16.86	
n-i-p TSC	Anti-Reflection Film/ITO/ZnO/SnO_2/PVK/D18-Cl/PEDOT:F/Au/ ZnO/PFN-Br/BHJ/2PACz-Cl/MoO_x/Ag		2.32	12.66	72.53	21.12	This Work
n-i-p PSC	ITO/ZnO/SnO_2/$CsPbI_2Br$ (~410 nm)/D18-Cl/MoO_x/Ag	1.89	1.40	15.83	78.46	17.44	
n-i-p OSC	ITO/ZnO/PFN-Br/D18-Cl:L8-BO:BTP-eC9/2PACz-Cl/MoO_x/Ag	1.43	0.925	25.18	71.00	16.54	
n-i-p TSC	Anti-Reflection Film/ITO/ZnO/SnO_2/PVK/D18-Cl/PEDOT:F/Au/ ZnO/PFN-Br/BHJ/2PACz-Cl/MoO_x/Ag		2.29	14.69	72.52	24.39	This Work
n-i-p PSC	ITO/ZnO/SnO_2/$CsPbI_2Br$ (~410 nm)/D18-Cl/MoO_x/Ag	1.89	1.40	15.83	78.46	17.44	
n-i-p OSC	ITO/ZnO/PFN-Br/PM6:L8-BO:BTP-eC9/2PACz-Cl/MoO_x/Ag	1.41	0.869	25.63	75.50	16.82	
n-i-p TSC	Anti-Reflection Film/ITO/ZnO/SnO_2/PVK/D18-Cl/PEDOT:F/Au/ ZnO/PFN-Br/BHJ/2PACz-Cl/MoO_x/Ag		2.23	14.98	75.75	25.34	This Work

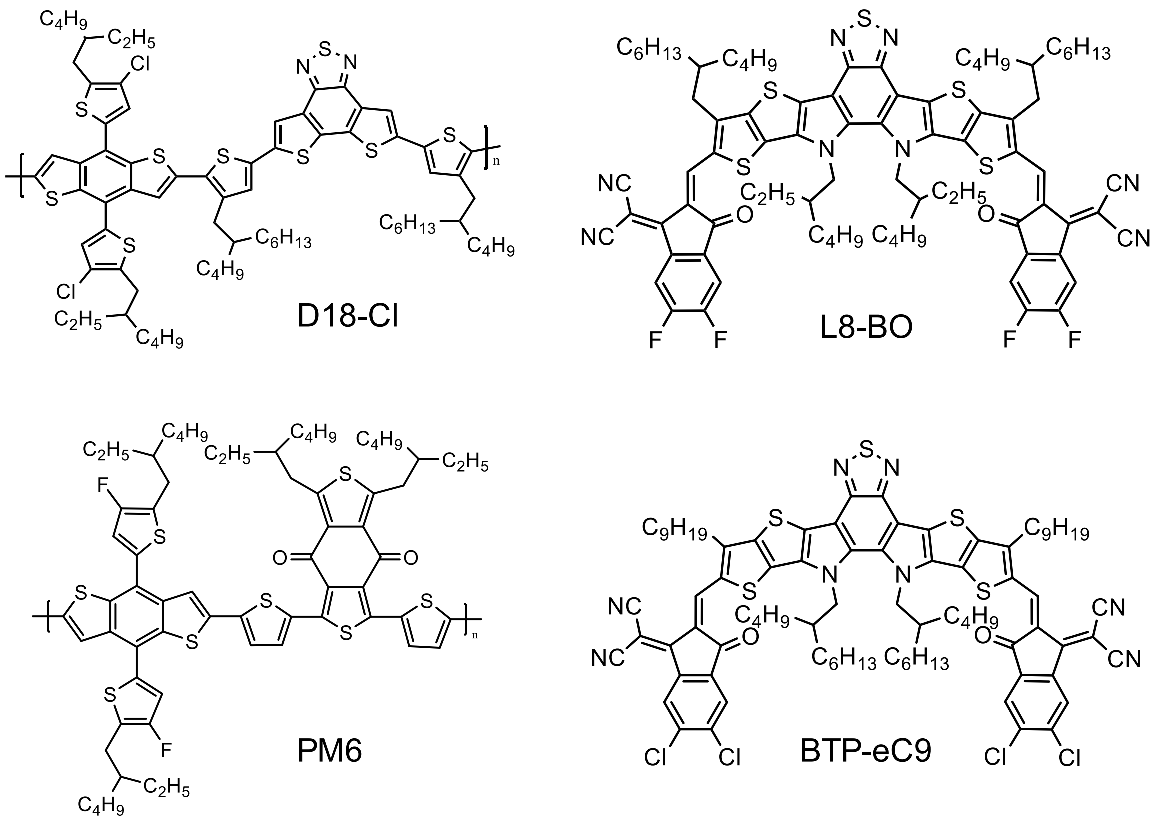

Figure S1. Chemical structures of the donors D18-Cl, PM6 and the acceptors L8-BO, BTP-eC9.

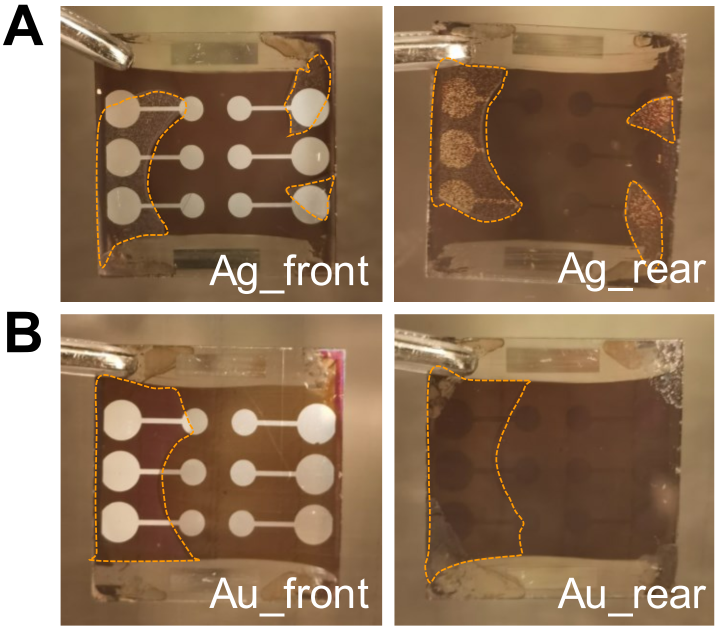

Figure S2. Images of the front and rear views of half-stacked-tandem devices (ITO/ETL/PVK/D18-Cl/MoO_x/Ag or Au/ZnO/PFN-Br/Ag) utilizing (A) Ag NPs and (B) Au NPs as the recombination sites. Due to incomplete coverage of D18-Cl (yellow dash range), the deposition of 1-nm Ag on the perovskite film directly caused a rapid degradation, while Au NPs exhibited a different positive behavior.

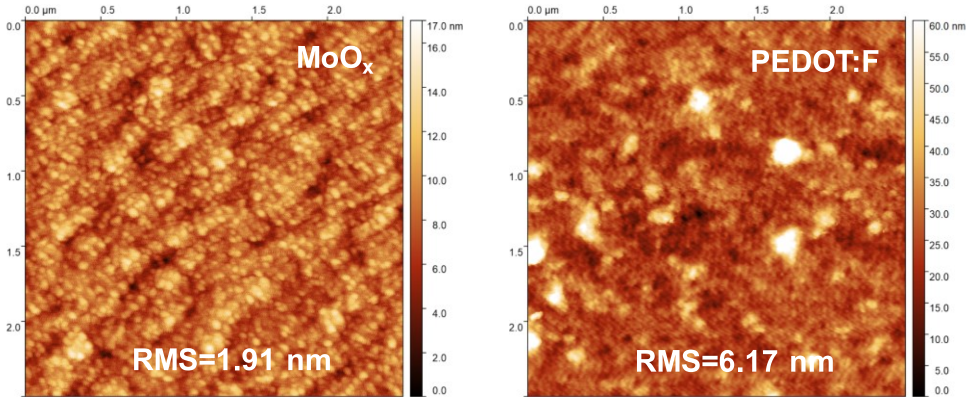

Figure S3. AFM images of MoO_x and PEDOT:F fabricated on the top of glass/D18-Cl.

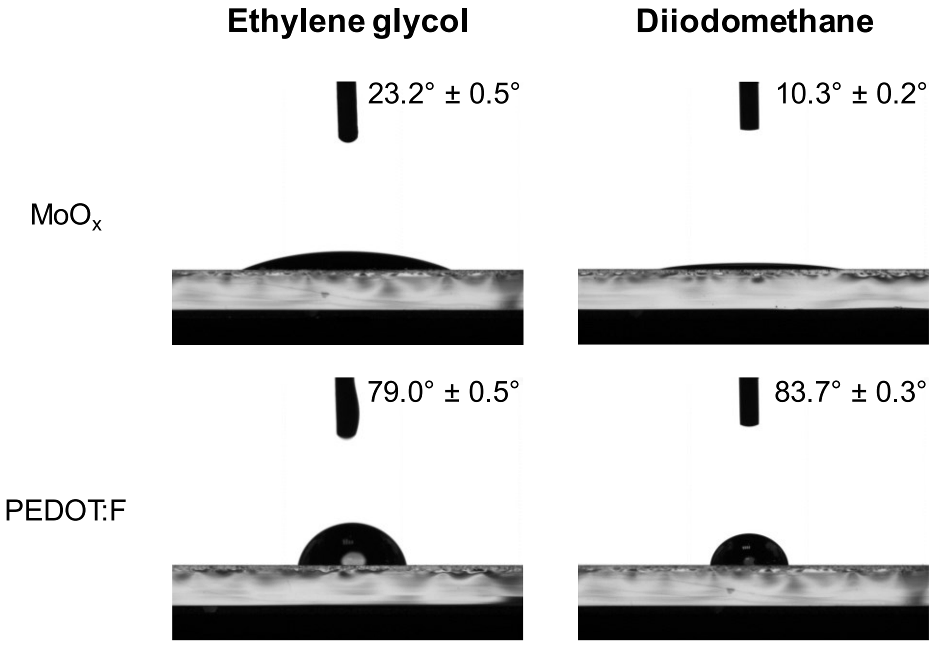

Figure S4. Contact angle measurements with ethylene glycol and diiodomethane on top of MoO_x and PEDOT:F films.

Table S2. Polar and dispersive surface energy components of test liquids and the average contact angles on MoO_x and PEDOT:F.

Liquid	γ_l(mJ/m^2)	γ_l^d(mJ/m^2)	γ_l^p(mJ/m^2)	ITO/MoO_x (°)	ITO/PEDOT:F (°)
water	72.8	21.8	51.0	7.6 ± 0.4	86.1 ± 0.2
ethylene glycol	48.0	29.0	19.0	23.2 ± 0.5	79.0 ± 0.5
diiodomethane	50.8	50.8	0.0	10.3 ± 0.2	83.7 ± 0.3

Table S3. The area parameters for the calculation of surface free energy through Wenzel model and the calculated results. The projected area and actual surface area of substrates are extracted from AFM images.

Substrate	Projected area (μm^2)	Actual surface area (μm^2)	*r*	γ_s^p (mJ/m^2)	γ_s^d (mJ/m^2)	γ_s (mJ/m^2)
MoO_x	6.25	6.676	1.068	28.02	0.30	28.32
PEDOT:F	6.25	7.414	1.186	5.26	1.67	6.93

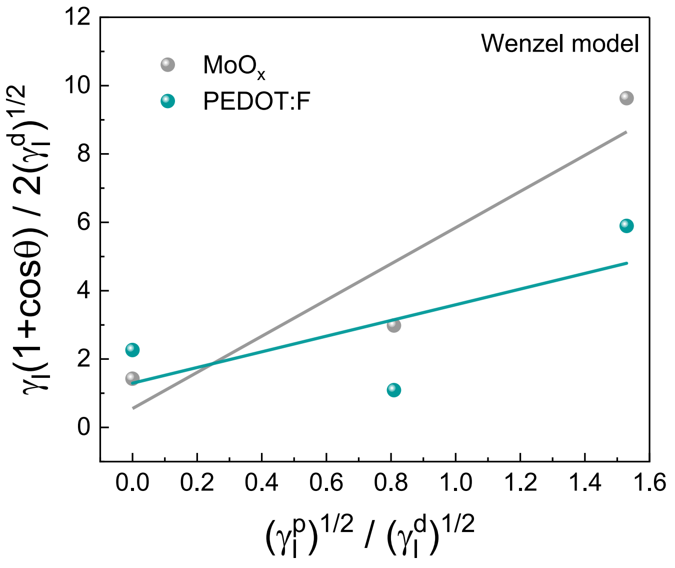

Figure S5. Wenzel plots for calculating γ_s^p and γ_s^d of MoO_x and PEDOT:F.

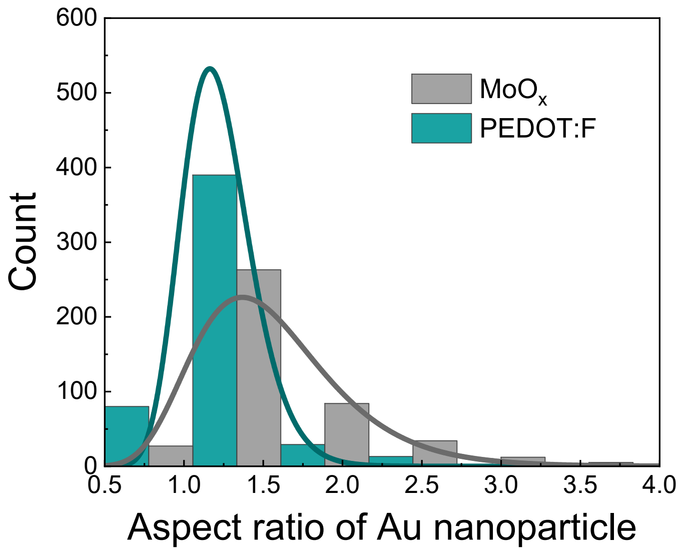

Figure S6. Aspect ratios of 1-nm Au NPs on MoO_x and PEDOT:F, which were extracted from SEM images in Figures 1D-E.

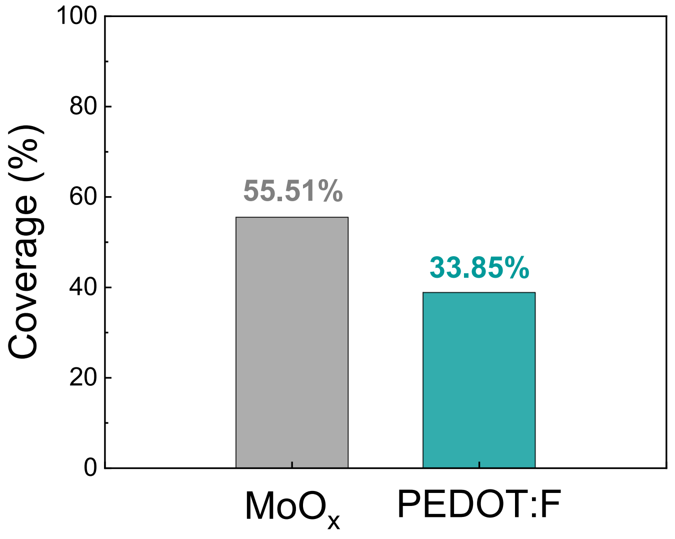

Figure S7. The statistics coverage of 1-nm Au NPs on the MoO_x and PEDOT:F films from SEM images in Figures 1D-E in the main text.

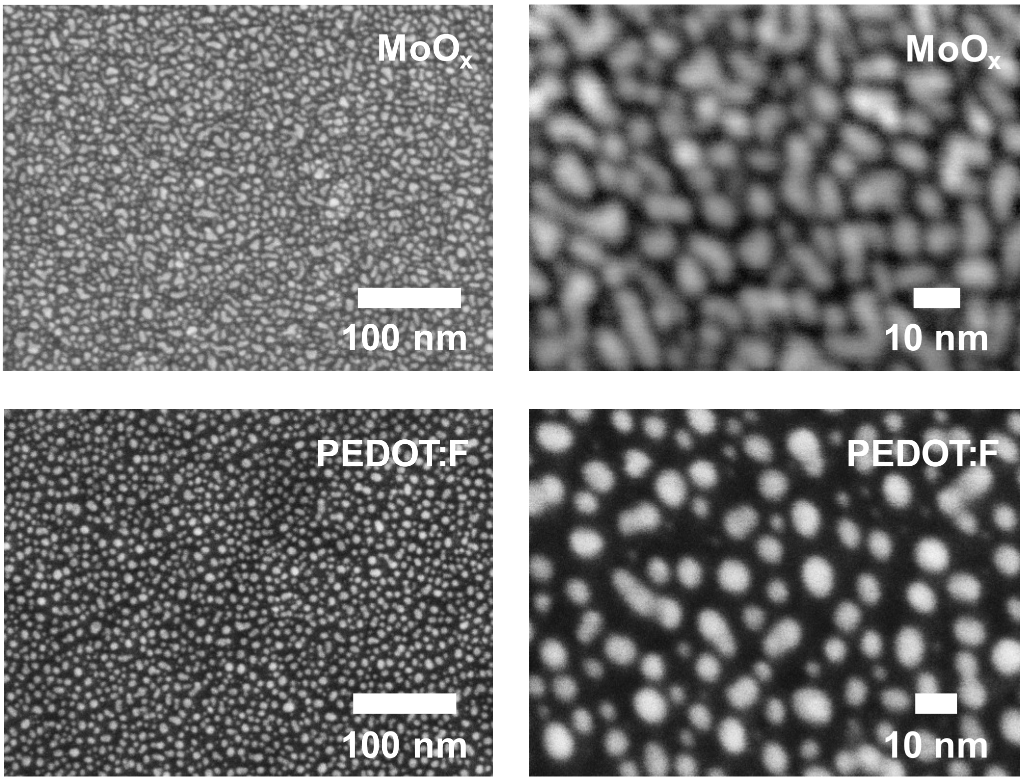

Figure S8. SEM images of 1-nm Au NPs on the MoO_x and PEDOT:F, captured under various magnifications. Here, the samples for SEM measurement were based on ITO/D18-Cl/MoO_x or PEDOT:F/1-nm Au structure. The deposition rate of Au NPs was 0.02 Å/s.

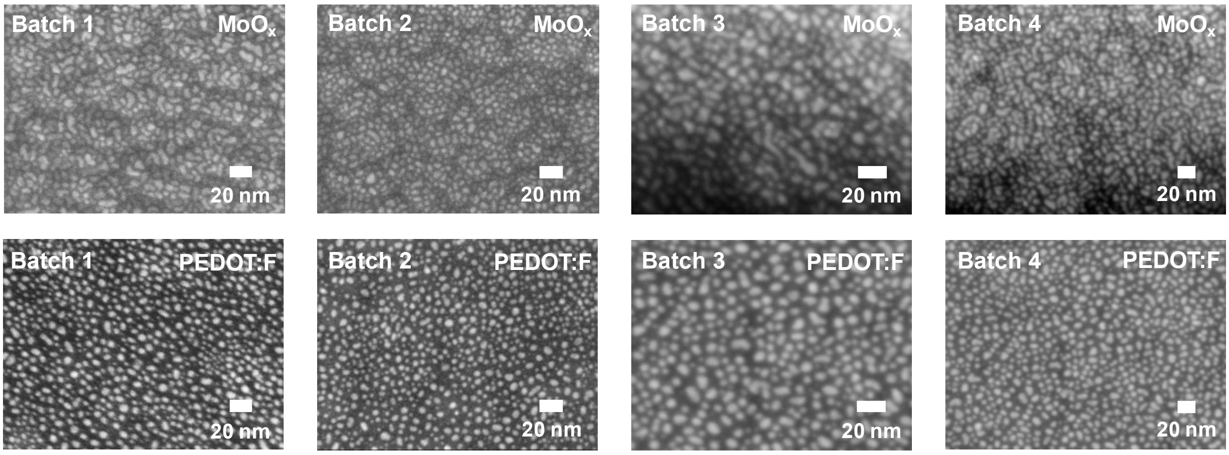

Figure S9. SEM images of 1-nm Au NPs on the MoO_x and PEDOT:F. The samples from Batch 1 and Batch 2 were based on ITO/MoO_x or PEDOT:F/1-nm Au. The samples from Batch 3 and Batch 4 were based on ITO/ETL/PVK/D18-Cl/MoO_x or PEDOT:F/1-nm Au. The deposition rate of Au NPs was 0.02 Å/s.

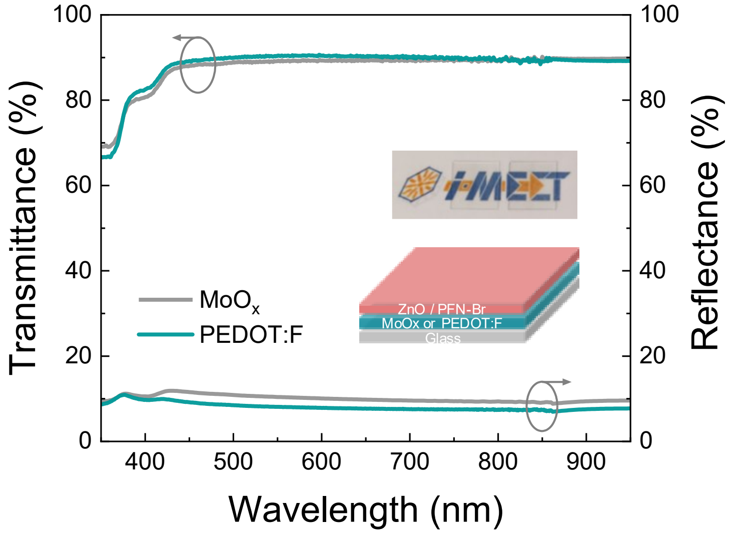

Figure S10. Optical characteristics (total transmittance and reflectance) of glass/MoO$_x$ or PEDOT:F/ZnO/PFN-Br with an inset of their photograph (left: MoO$_x$, right: PEDOT:F).

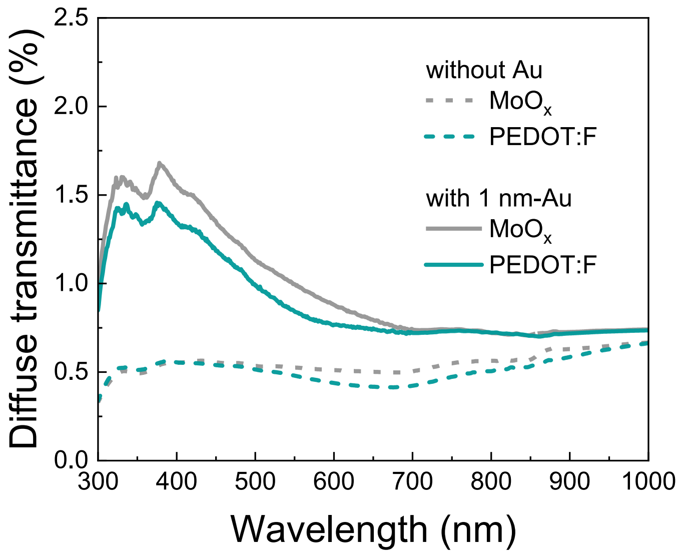

Figure S11. Diffuse transmittance of ICLs (glass/MoO$_x$ or PEDOT:F/with or without Au/ZnO/PFN-Br).

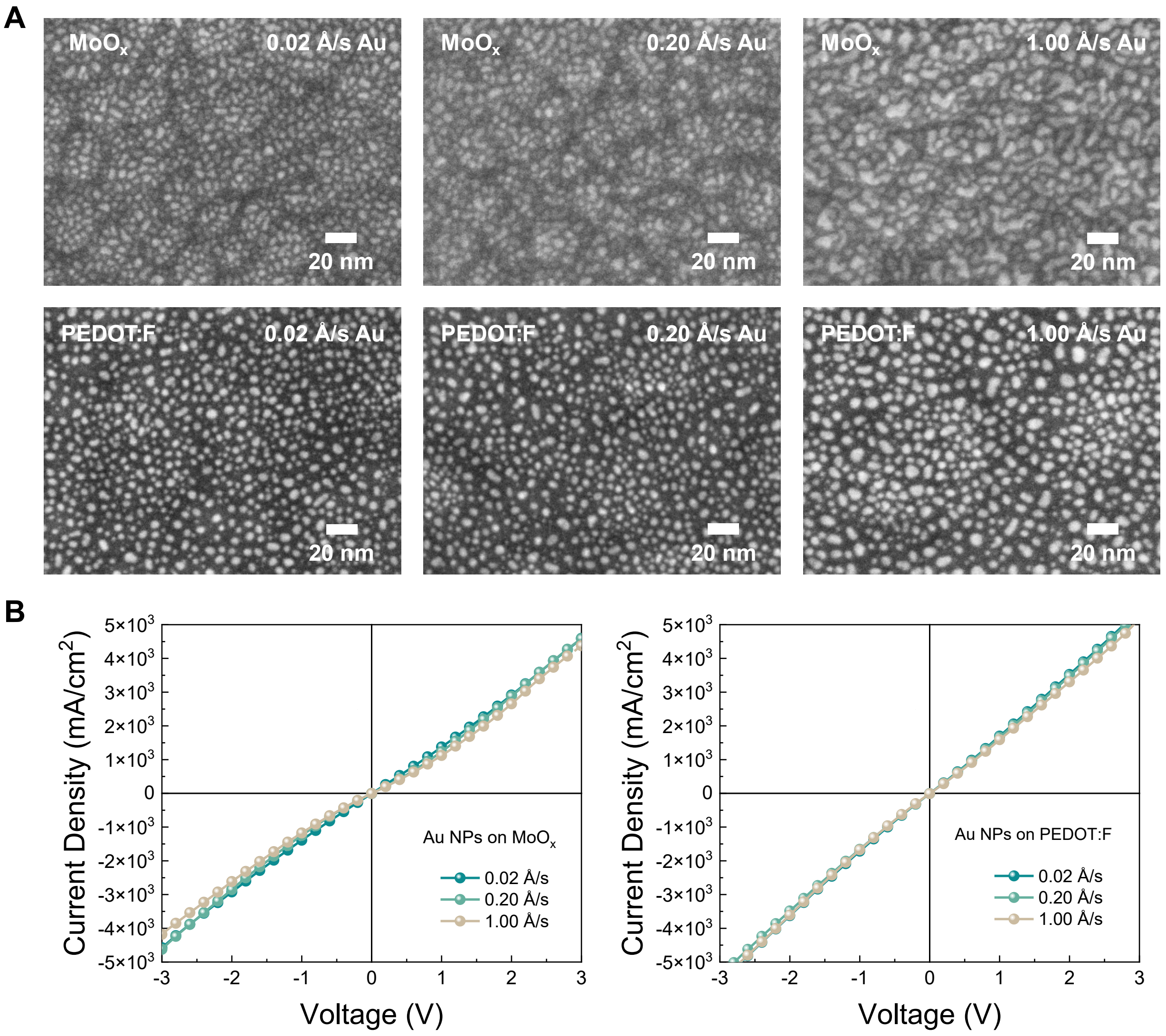

Figure S12. (A) SEM images of 1-nm Au NPs deposited on MoO_x and PEDOT:F at different evaporation rates (0.02 Å/s, 0.20 Å/s, and 1.00 Å/s). Here, all samples for SEM measurement were based on ITO/MoO_x or PEDOT:F/1-nm Au structure. (B) The corresponding *J-V* curves of ICLs (ITO/MoO_x or PEDOT:F/Au/ZnO/PFN-Br/Ag) with different deposition rates of Au (0.02 Å/s, 0.20 Å/s, and 1.00 Å/s).

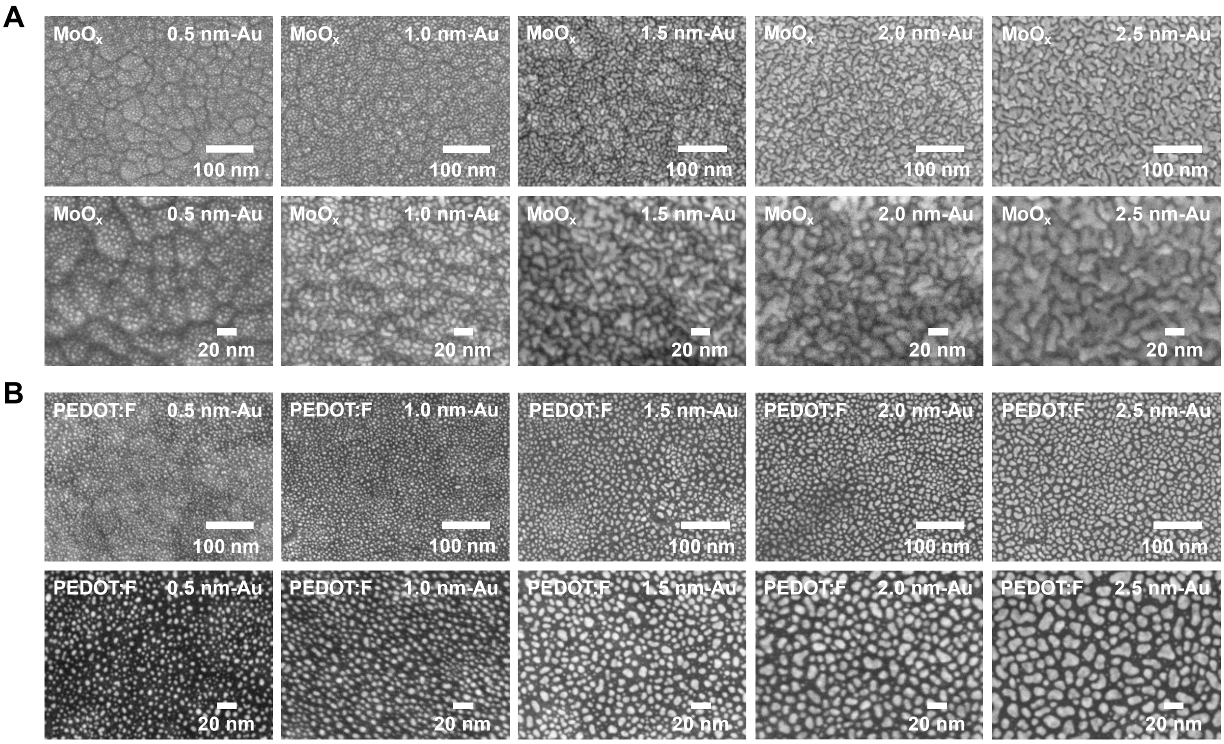

Figure S13. SEM images at 250,000x and 500,000x magnifications of Au NPs deposited on (A) MoO_x and (B) PEDOT:F with varying thicknesses (0.5 nm, 1.0 nm, 1.5 nm, 2.0 nm, 2.5 nm). Here, all samples for SEM measurement were based on ITO/MoO_x or PEDOT:F/Au structure.

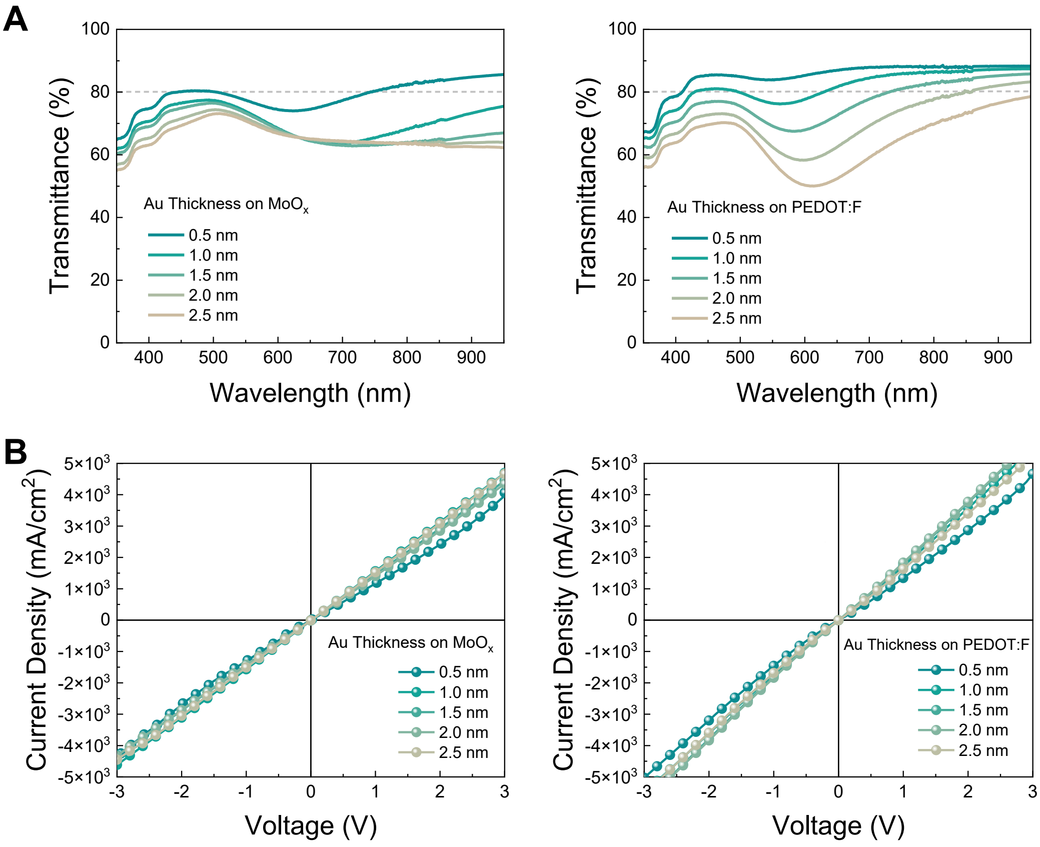

Figure S14. (A) The total transmittance spectra of ICLs (glass/MoO_x or PEDOT:F/Au/ZnO/PFN-Br) with varying Au thicknesses. (B) The corresponding *J-V* curves of ICLs (ITO/MoO_x or PEDOT:F/Au/ZnO/PFN-Br/Ag) with varying Au thicknesses.

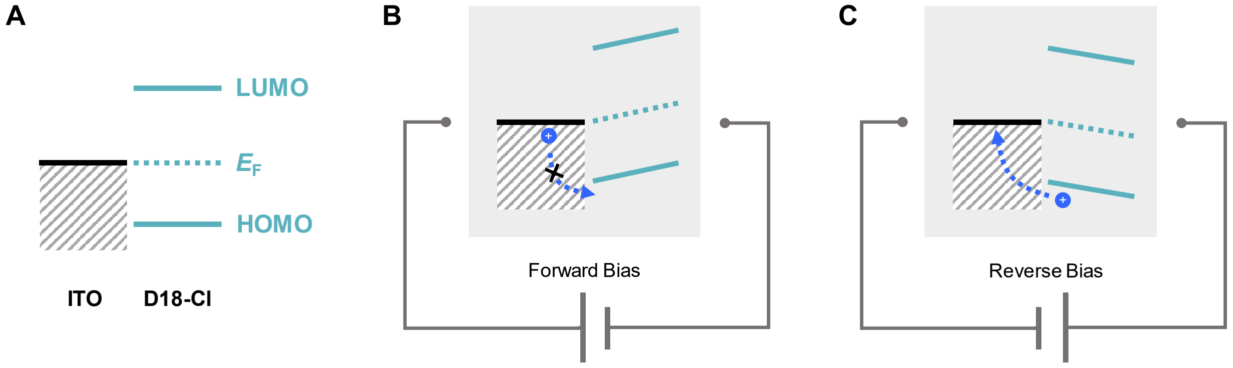

Figure S15. (A) Energy level diagram of ITO and D18-Cl at zero bias. (B) Energy level diagram of ITO and D18-Cl under forward bias. (C) Energy level diagram of ITO and D18-Cl under reverse bias.

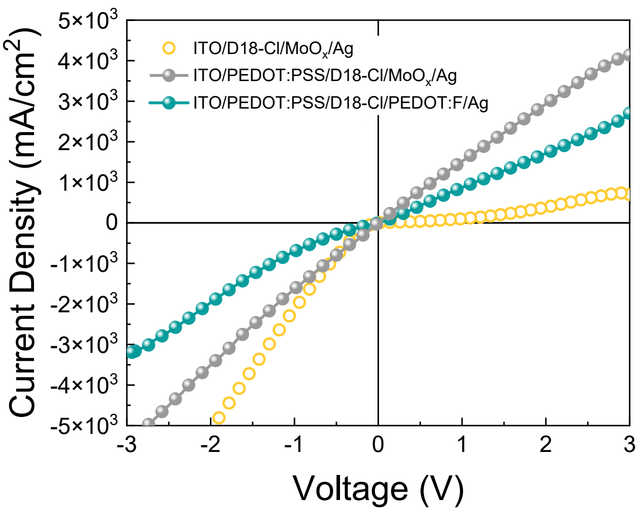

Figure S16. Dark *J-V* curves of the devices with different architectures: ITO/D18-Cl/MoO_x/Ag, ITO/PEDOT:PSS/D18-Cl/MoO_x/Ag, and ITO/PEDOT:PSS/D18-Cl/PEDOT:F/Ag. First quadrant: hole injection from ITO.

Energy barrier between ITO and D18-Cl

Figure S15A shows the energy level diagram between ITO and D18-Cl at zero bias. It is evident that D18-Cl film exhibits a deeper HOMO than the work function of ITO. As illustrated in Figures S15B and S15C, applying a forward bias across the ITO/D18-Cl stack results in an inability to inject holes from ITO due to a substantial energy barrier between ITO and the HOMO of D18-Cl. Conversely, with reverse bias, holes can be efficiently transferred from D18-Cl to ITO.

This behavior is reflected evidently in the *J-V* curve of the ITO/D18-Cl/MoO_x/Ag device, as shown in Figure S16. When ITO is the positive electrode (first quadrant), current injection is notably suppressed. In contrast, with Ag as the positive electrode (third quadrant), holes are effectively injected, leading to a significant linearly increasing current. Introducing a PEDOT:PSS layer between D18-Cl and ITO can mitigate this issue, resulting in *J-V* characteristics for ITO/PEDOT:PSS/D18-Cl/MoO_x/Ag that display linear, quasi-ohmic contact behavior. However, the deep HOMO of D18-Cl does not impact the performance of single-junction perovskite devices with an n-i-p architecture. For $CsPbI_2Br$ perovskite, the conduction band (*e.g.* 5.78 eV) is considerably deeper than the HOMO of D18-Cl, eliminating any energy barrier for hole transfer. Consequently, photo-generated holes in $CsPbI_2Br$ perovskite layer are efficiently extracted and transferred to Ag electrode via D18-Cl HTL.

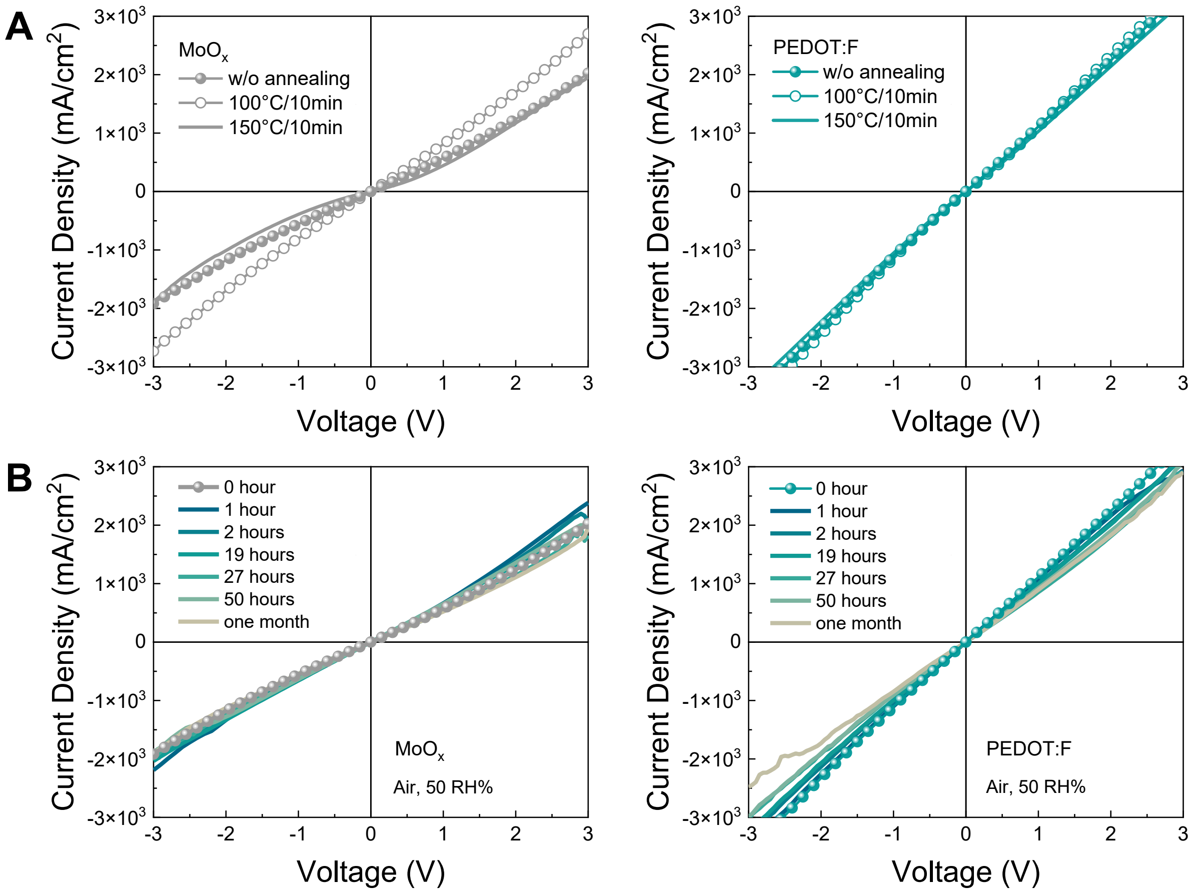

Figure S17. *J-V* curves of the ICLs-only devices (ITO/MoO_x or PEDOT:F/Au/ZnO/PFN-Br/Ag) after (A) thermal annealing and (B) exposure to air.

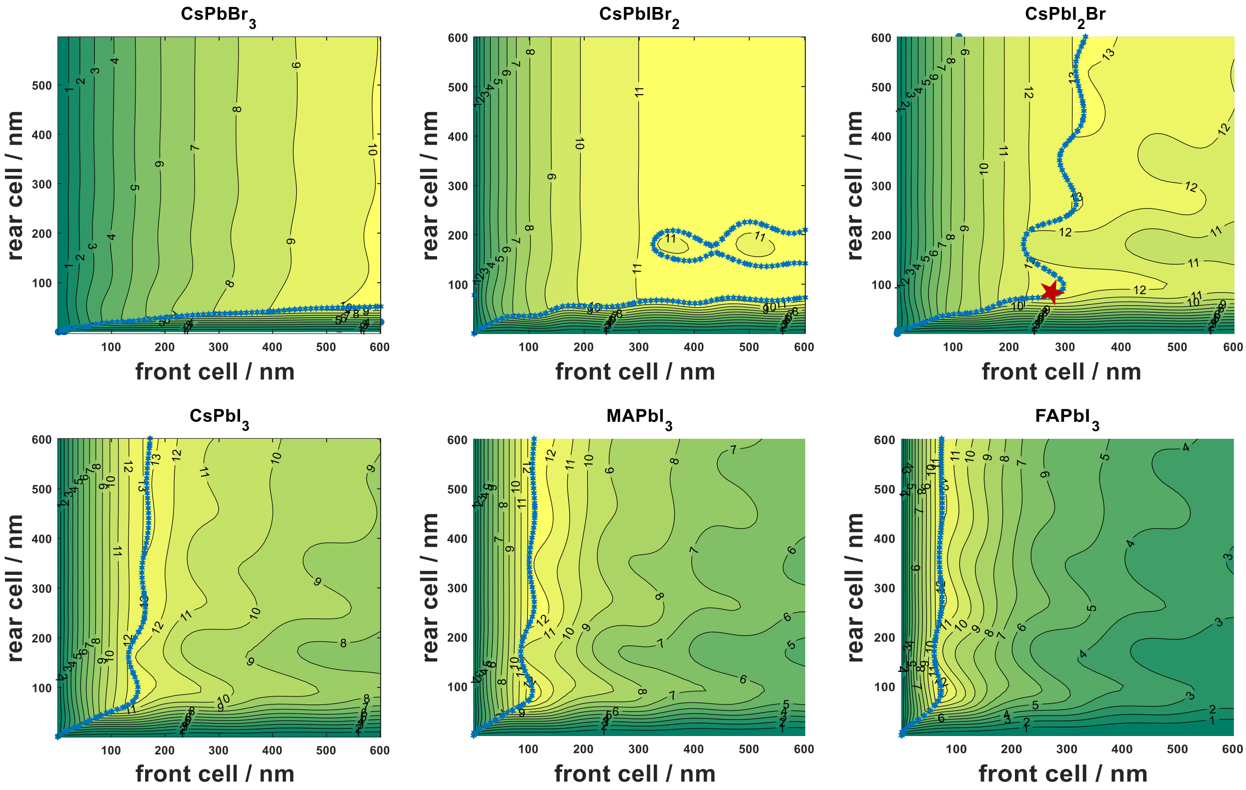

Figure S18. Simulated current-density as a function of the variable thicknesses of the front perovskite cell with varied bandgaps and D18-Cl:L8-BO rear cell with a bandgap of 1.46 eV. The blue curve indicates that the current is matched theoretically.

Table S4. Efficiency limits of 2-terminal perovskite-organic tandem photovoltaics based on D18-Cl:L8-BO rear cell with different front cell bandgaps. The achievable maximum thicknesses (blue color) of high-quality PVK and BHJ films in the experiments are extracted from the literature (ref. 24-30).

Front Cell	Bandgap (eV)	$J_{SC,TSC}$ (mA/cm^2)	PVK Thickness (nm)	BHJ Thickness (nm)	Front Cell $V_{OC,PSC}$ (V)	Rear Cell $V_{OC,OSC}$ (V)	Tandem $V_{OC,TSC}$ (V)	Fill Factor	Efficiency (%)
$CsPbBr_3$	2.340	10.48 (10.48)	501 (501)	51 (51)	1.702 [24]	0.938	2.640	0.80	22.13 (22.13)
$CsPbIBr_2$	2.120	11.76 (10.10)	591 (251)	71 (51)	1.327 [25]	0.938	2.265	0.80	21.31 (18.30)
$CsPbI_2Br$	1.893	13.31 (12.81)	341 (284)	601 (91)	1.450 [27]	0.938	2.388	0.80	25.43 (24.47)
$CsPbI_3$	1.746	13.40 (12.69)	171 (149)	601 (91)	1.330 [28]	0.938	2.268	0.80	24.31 (23.02)
$MAPbI_3$	1.590	12.93 (12.59)	111 (111)	451 (91)	1.130 [29]	0.938	2.068	0.80	21.39 (20.83)
$FAPbI_3$	1.550	12.74 (12.74)	72 (72)	91 (91)	1.189 [30]	0.938	2.127	0.80	21.68 (21.68)

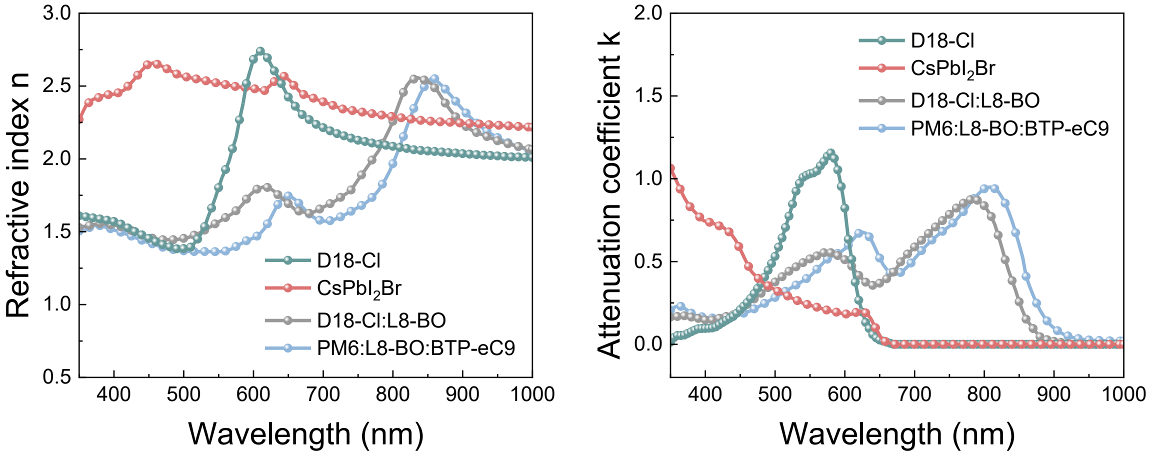

Figure S19. The optical constants of D18-Cl, $CsPbI_2Br$, D18-Cl:L8-BO and PM6:L8-BO:BTP-eC9 used in our optical simulation.

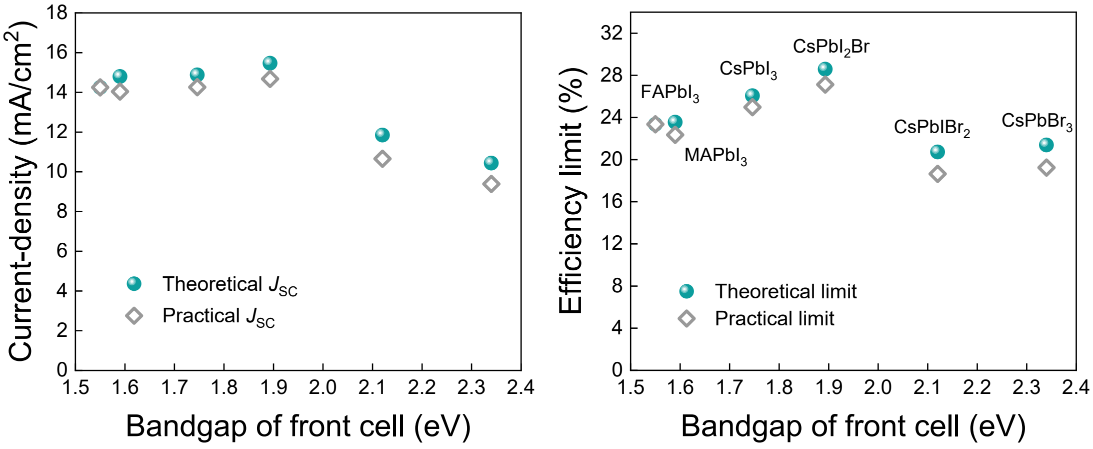

Figure S20. Current-density and efficiency limits of 2T TSCs based on high-performance rear cell (1.41 eV, PM6:L8-BO:BTP-eC9) with different front cell bandgaps.

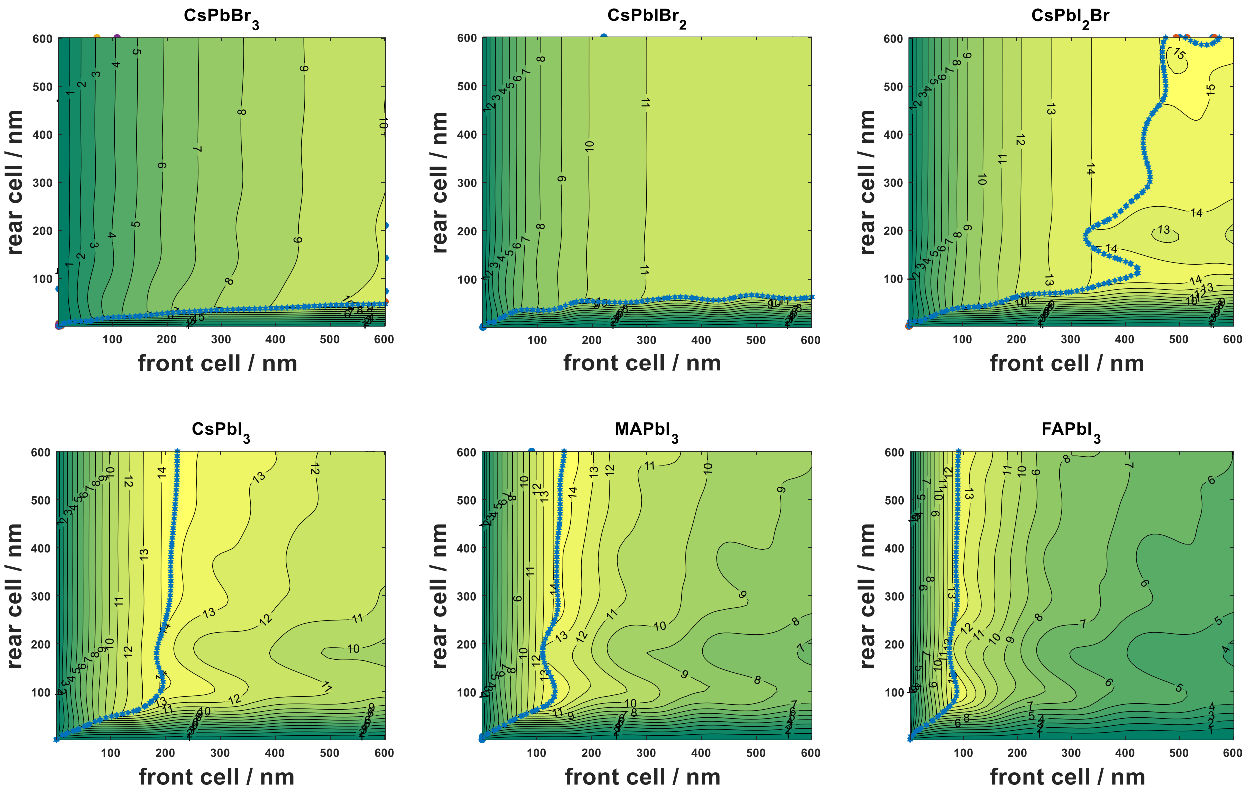

Figure S21. Simulated current-density as a function of the variable thicknesses of the front perovskite cell with varied bandgaps and PM6:L8-BO:BTP-eC9 rear cell with a bandgap of 1.41 eV. The blue curve indicates that the current is matched theoretically.

Table S5. Efficiency limits of 2-terminal perovskite-organic tandem photovoltaics based on PM6:L8-BO:BTP-eC9 rear cell with different front cell bandgaps. The achievable maximum thicknesses (blue color) of high-quality PVK and BHJ films in the experiments are extracted from the literature (ref. 24-30).

Front Cell	Bandgap (eV)	$J_{SC,TSC}$ (mA/cm^2)	PVK Thickness (nm)	BHJ Thickness (nm)	Front Cell $V_{OC,PSC}$ (V)	Rear Cell $V_{OC,OSC}$ (V)	Tandem $V_{OC,TSC}$ (V)	Fill Factor	Efficiency (%)
$CsPbBr_3$	2.340	10.44 (9.39)	601 (501)	46 (44)	1.702	0.860	2.562	0.80	21.40 (19.25)
$CsPbIBr_2$	2.120	11.85 (10.66)	601 (251)	63 (52)	1.327	0.860	2.187	0.80	20.73 (18.65)
$CsPbI_2Br$	1.893	15.47 (14.68)	571 (423)	601 (111)	1.450	0.860	2.310	0.80	28.59 (27.13)
$CsPbI_3$	1.746	14.88 (14.26)	221 (195)	601 (121)	1.330	0.860	2.190	0.80	26.07 (24.98)
$MAPbI_3$	1.590	14.80 (14.04)	151 (133)	601 (101)	1.130	0.860	1.990	0.80	23.56 (22.35)
$FAPbI_3$	1.550	14.25 (14.25)	86 (86)	91 (91)	1.189	0.860	2.049	0.80	23.36 (23.36)

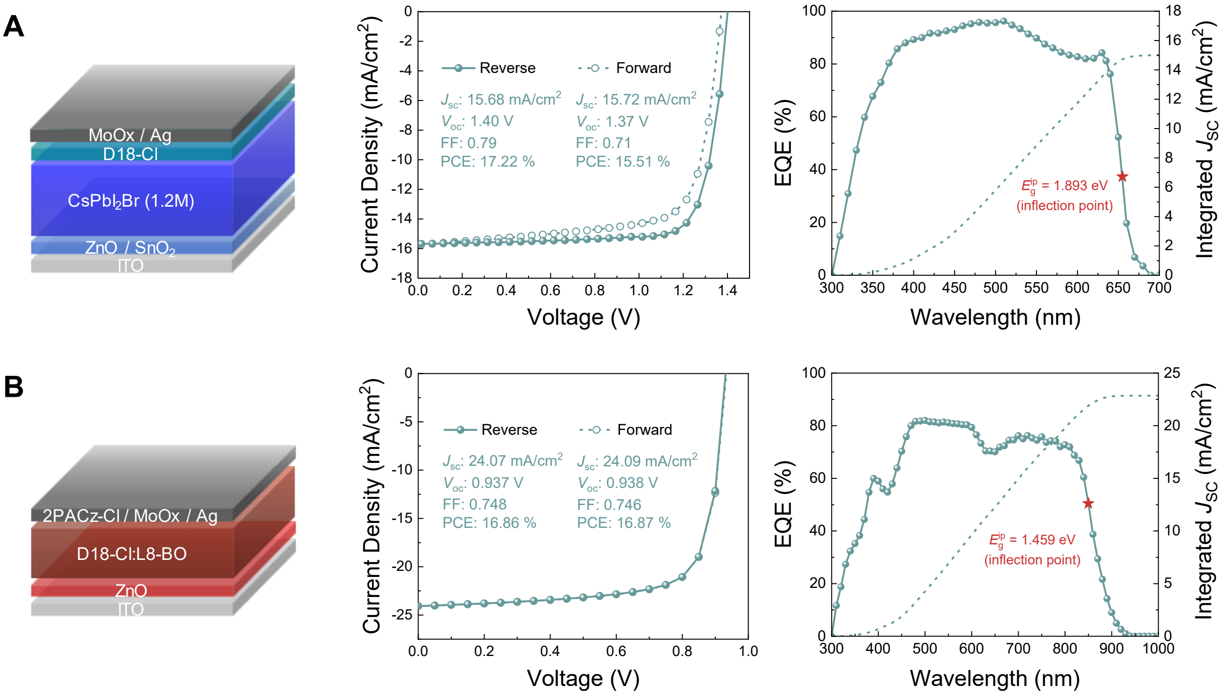

Figure S22. Device architectures, champion performance and the corresponding EQE spectra of (A) single-junction $CsPbI_2Br$ PSCs and (B) single-junction D18-Cl:L8-BO OSCs.

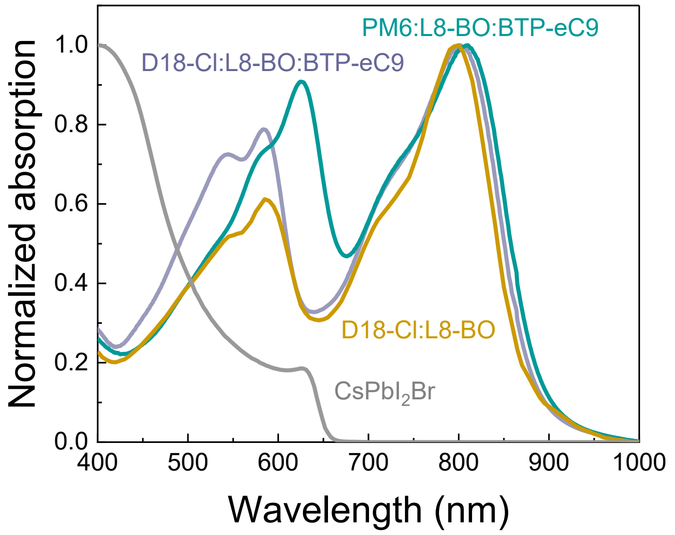

Figure S23. Normalized absorption spectra of $CsPbI_2Br$, PM6:L8-BO:BTP-eC9, D18-Cl:L8-BO and D18-Cl:L8-BO:BTP-eC9 films.

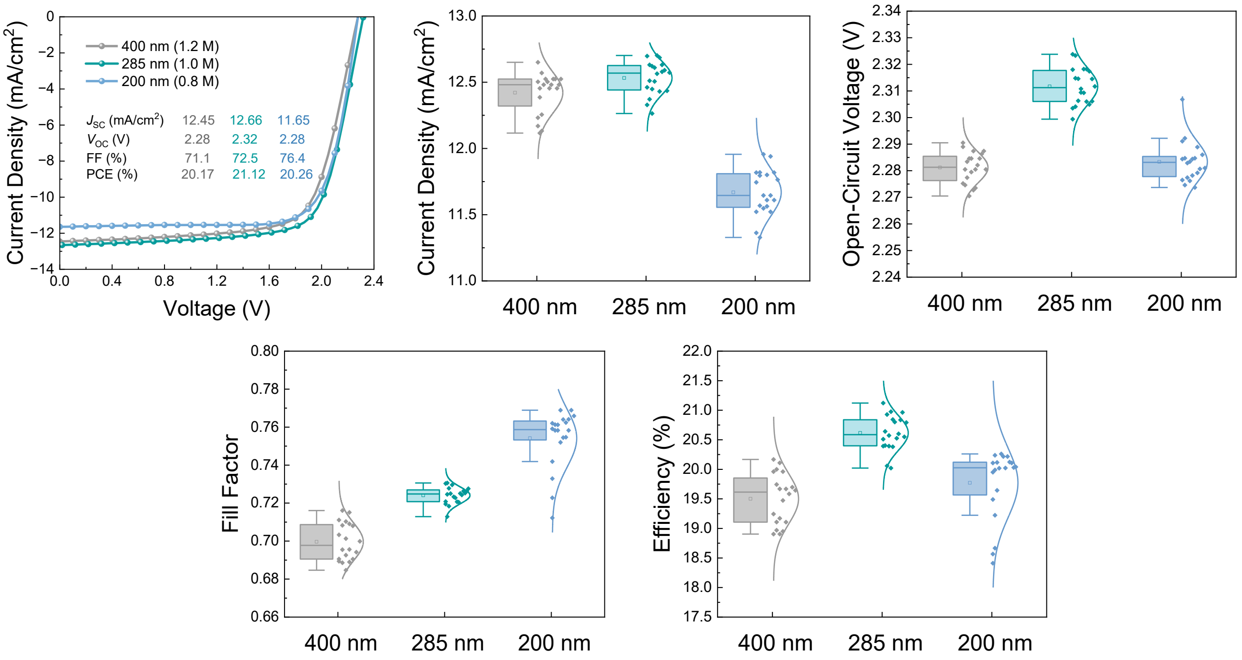

Figure S24. Device performance of $CsPbI_2Br$/D18-Cl:L8-BO TSCs under different thicknesses of front cell, employing the PEDOT:F-based ICLs. The parameters were obtained from two batches (20 devices).

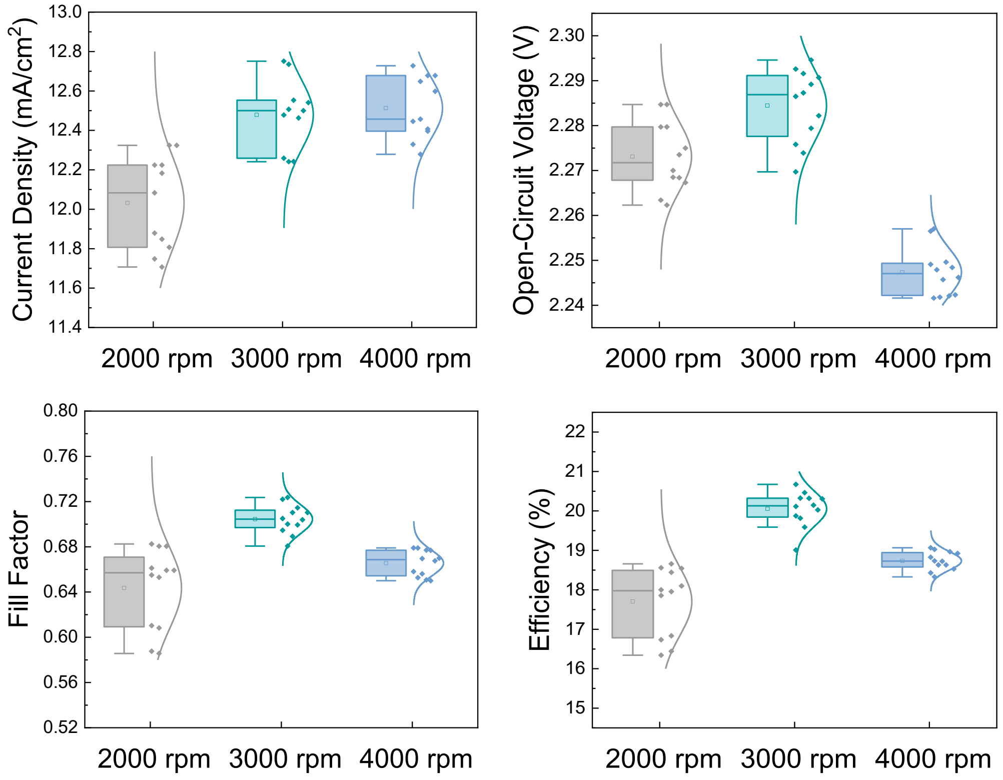

Figure S25. Statistical box plots for device parameters of $CsPbI_2Br$/D18-Cl:L8-BO TSCs under different thicknesses of rear cell, employing the PEDOT:F-based ICLs. Here, 3000 rpm was the optimal condition, corresponding to a film thickness of ~93 nm. The parameters were obtained from one batch (12 devices, a less optimal batch).

Table S6. Device parameters of champion $CsPbI_2Br$-D18-Cl:L8-BO TSCs with MoO_x-based ICLs and PEDOT:F-based ICLs, and the corresponding sub-cells.

Device		V_{OC} (V)	J_{SC} (mA/cm^2)	FF (%)	PCE (%)
TSC (w ARF)	MoO_x-based ICLs	2.27	11.10	78.17	19.69
	PEDOT:F-based ICLs	2.32	12.66	72.53	21.12
Front (w/o ARF)		1.40	13.84	82.54	15.98
Rear (w/o ARF)		0.937	24.07	74.80	16.86

Table S7. Device performance of champion $CsPbI_2Br$-D18-Cl:L8-BO TSC with the PEDOT:F-based ICLs under reverse and forward scans.

Scan Direction	V_{OC} (V)	J_{SC} (mA/cm^2)	FF (%)	PCE (%)
Reverse	2.32	12.66	72.53	21.12
Forward	2.28	12.69	69.29	19.98

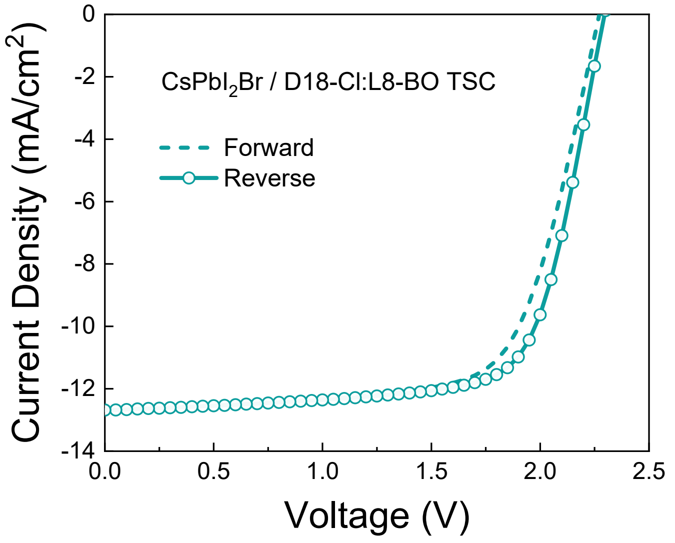

Figure S26. *J-V* curves of champion $CsPbI_2Br$/D18-Cl:L8-BO TSCs employing PEDOT:F under reverse and forward scans.

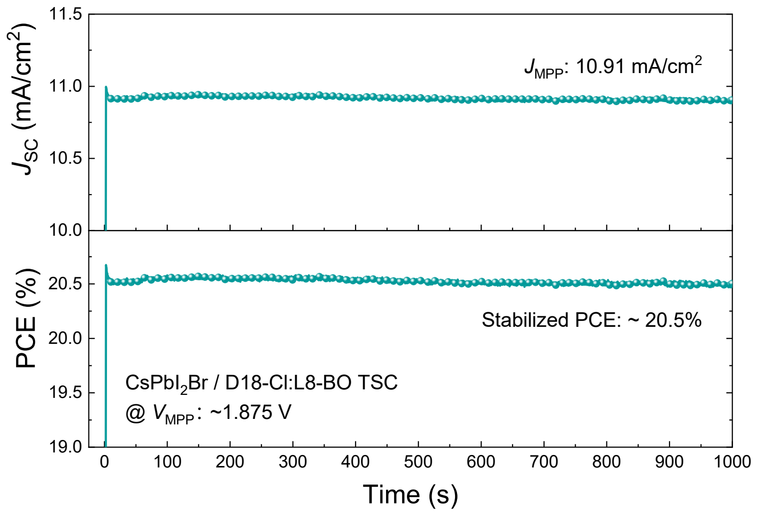

Figure S27. Corresponding MPP tracking of champion CsPbI$_2$Br/D18-Cl:L8-BO TSC under solar simulator in air.

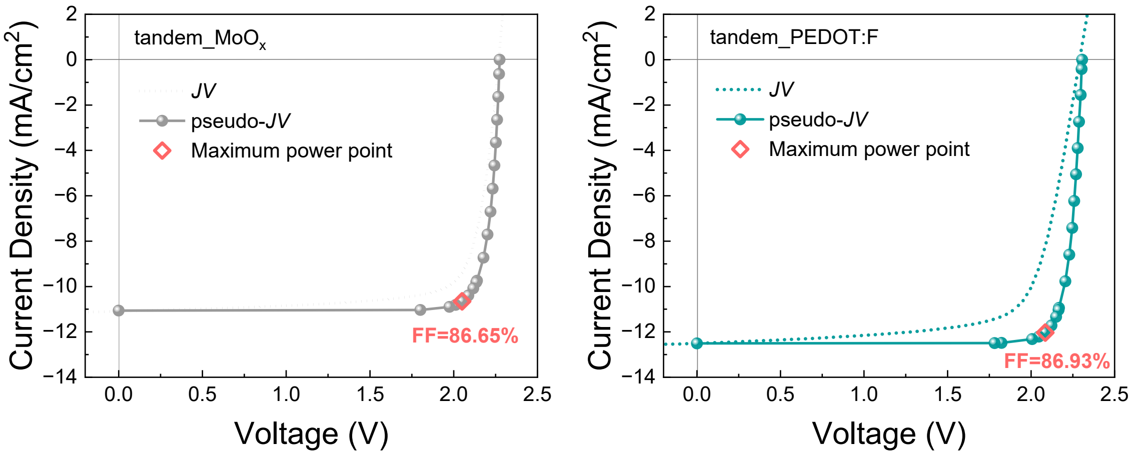

Figure S28. The pseudo-*JV* curves of CsPbI$_2$Br/D18-Cl:L8-BO TSCs employing MoO$_x$ and PEDOT:F, which are created from intensity-dependent *J*-*V* measurements.

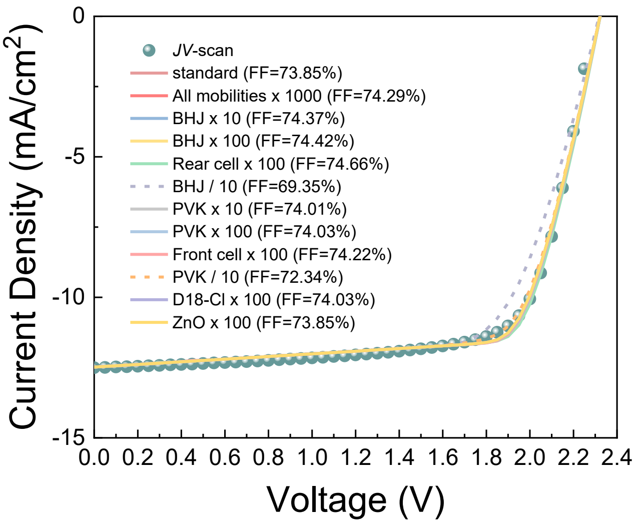

Figure S29. Simulated *JV*-curves of $CsPbI_2Br$/D18-Cl:L8-BO TSCs with PEDOT:F-based ICLs, using Setfos software. Based on the standard simulation (pink) with a FF of 73.85%, we increased the carrier mobilities of the individual layers to lower the charge transport losses. The graph shows that a 10/100/1000-fold increase of the mobility has no effect on the obvious FF.

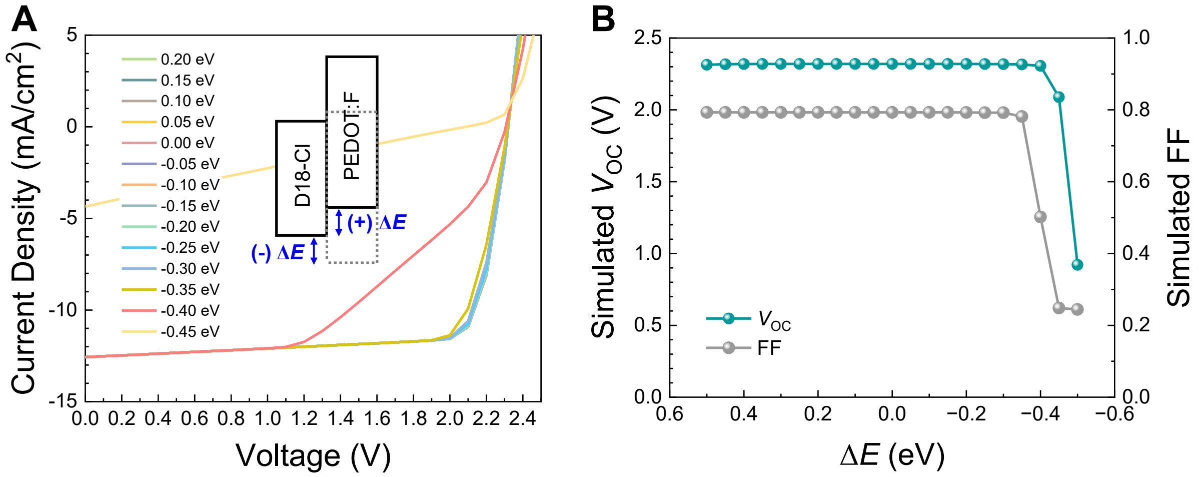

Figure S30. (A) The impact of energy difference (ΔE) between D18-Cl and PEDOT:F on the *JV*-curves of $CsPbI_2Br$/D18-Cl:L8-BO TSCs with PEDOT:F-based ICLs, simulated using Setfos software. (B) Simulated V_{OC} and FF as a function of ΔE.

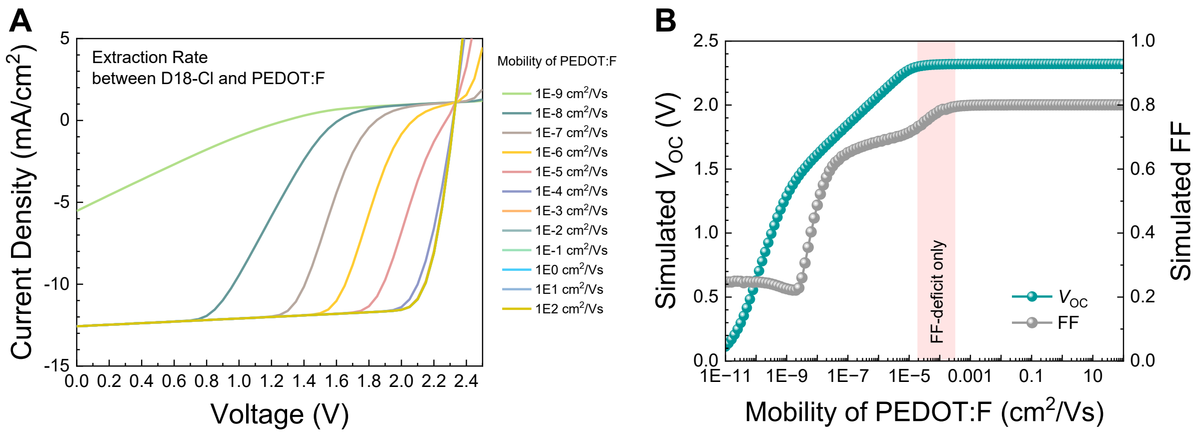

Figure S31. (A) The impact of the extraction rate from D18-Cl to PEDOT:F on the *JV*-curves of $CsPbI_2Br$/D18-Cl:L8-BO TSCs with PEDOT:F-based ICLs, simulated using Setfos software. Since the extraction rate value between the two layers cannot be directly adjusted in Setfos, we simulated a reduced extraction rate from D18-Cl to PEDOT:F by lowering the mobility of PEDOT:F. (B) Simulated V_{OC} and FF as a function of the extraction rate from D18-Cl to PEDOT:F.

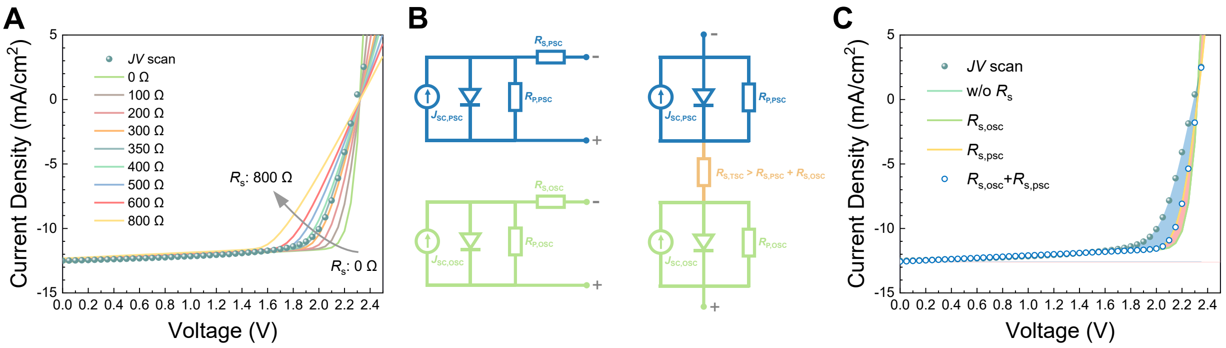

Figure S32. (A) The impact of varied series resistance (R_S) on *JV*-curves of $CsPbI_2Br$/D18-Cl:L8-BO TSCs with PEDOT:F-based ICLs, obtained from the Setfos simulation. (B) The equivalent circuits of single-junction PSC, single-junction OSC and P-O-TSC. (C) The simulated *JV*-curves of $CsPbI_2Br$/D18-Cl:L8-BO TSCs with PEDOT:F-based ICLs, based on $R_{S,PSC}$, $R_{S,OSC}$ and $R_{S,PSC}$+$R_{S,OSC}$. Here, $R_{S,PSC}$ and $R_{S,OSC}$ are the series resistances of single-junction PSC and single-junction OSC, respectively. It is evident that the series resistance introduced by front and rear cells has slightly adverse effect on tandem's FF (pink shade), suggesting that the FF value is primarily subject to the series resistance introduced by PEDOT:F-based ICLs (blue shade).

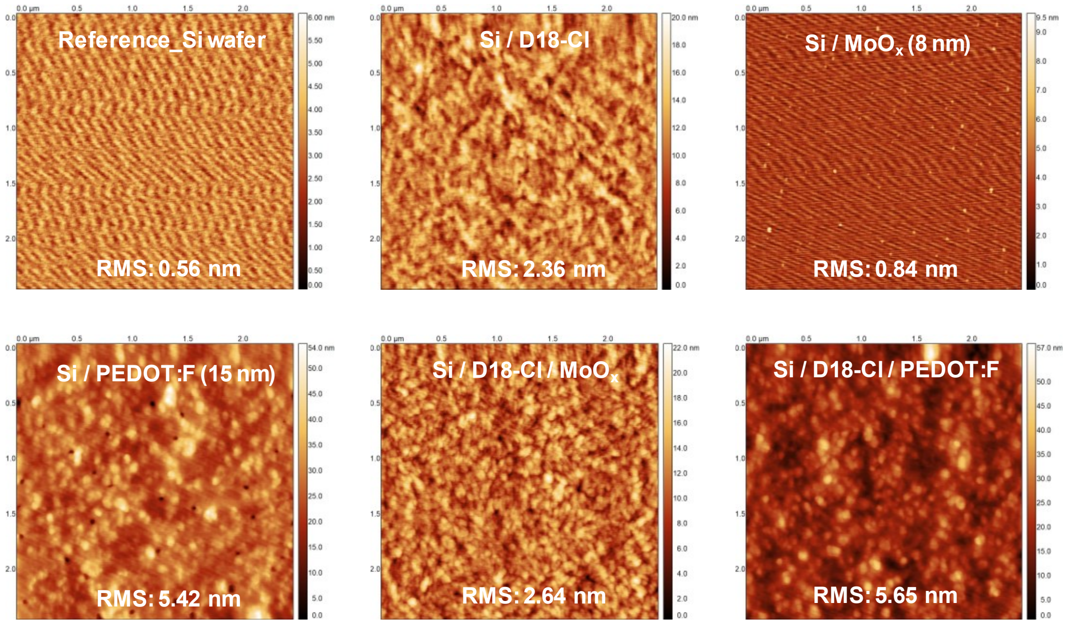

Figure S33. AFM images of silicon wafer (Si), Si/D18-Cl, Si/MoO_x, Si/PEDOT:F, Si/D18-Cl/MoO_x and Si/D18-Cl/PEDOT:F. The pure D18-Cl layer demonstrates a relatively low roughness of 2.36 nm, while the MoO_x layer exhibits negligible roughness. In contrast, the PEDOT:F layer shows substantial roughness, measuring 5.42 nm. These findings confirm that the pronounced roughness of PEDOT:F arises from its intrinsic material properties. We hypothesize that the highly rough PEDOT:F layer forms incomplete layer-to-layer contact with D18-Cl, potentially creating microscopic voids or gaps at the interface, which adversely impacts the performance of PEDOT:F-based P-O-TSCs.

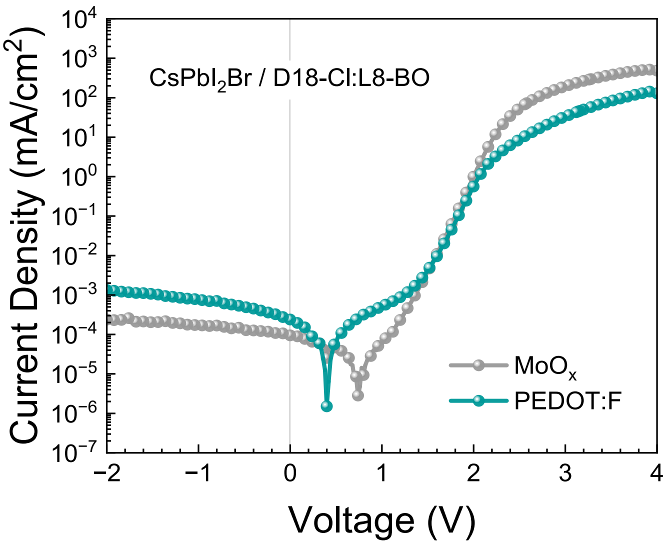

Figure S34. Dark *J-V* curves of $CsPbI_2Br$/D18-Cl:L8-BO TSCs employing MoO_x and PEDOT:F.

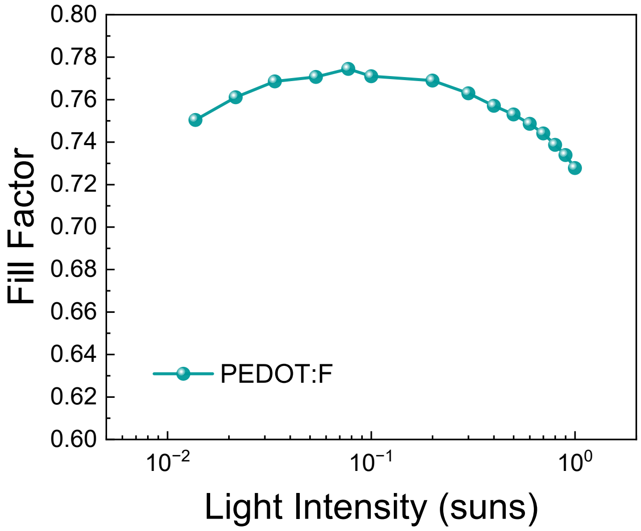

Figure S35. Light-intensity dependent fill factor of $CsPbI_2Br$/D18-Cl:L8-BO TSCs employing PEDOT:F.

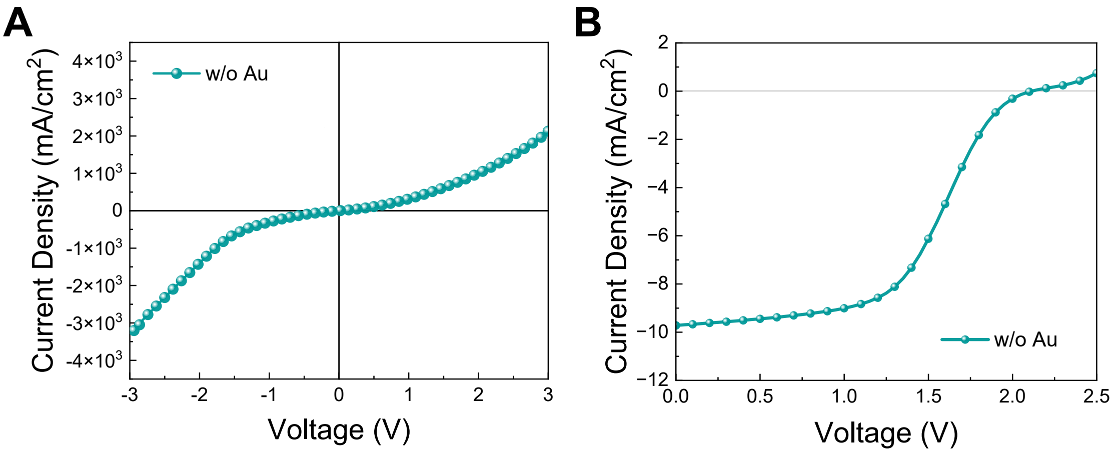

Figure S36. *J-V* characteristics of (A) ICL-only device with a structure of ITO/MoO_x/ZnO/PFN-Br/Ag and (B) tandem cells with MoO_x/ZnO/PFN-Br ICLs.

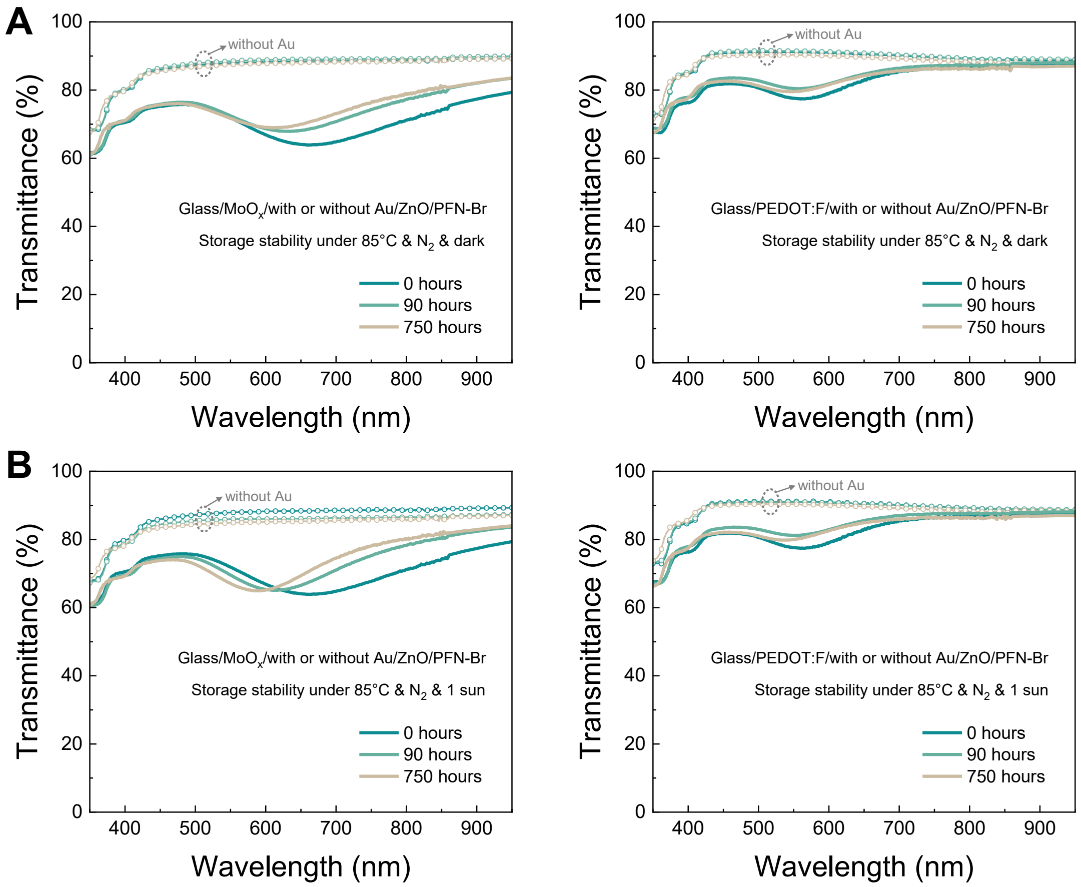

Figure S37. Stability of total transmittance of Glass/MoO$_x$ or PEDOT:F/with or without Au/ZnO/PFN-Br stacks under (A) 85°C & N_2 & dark condition, and (B) 85°C & N_2 & 1 sun condition.

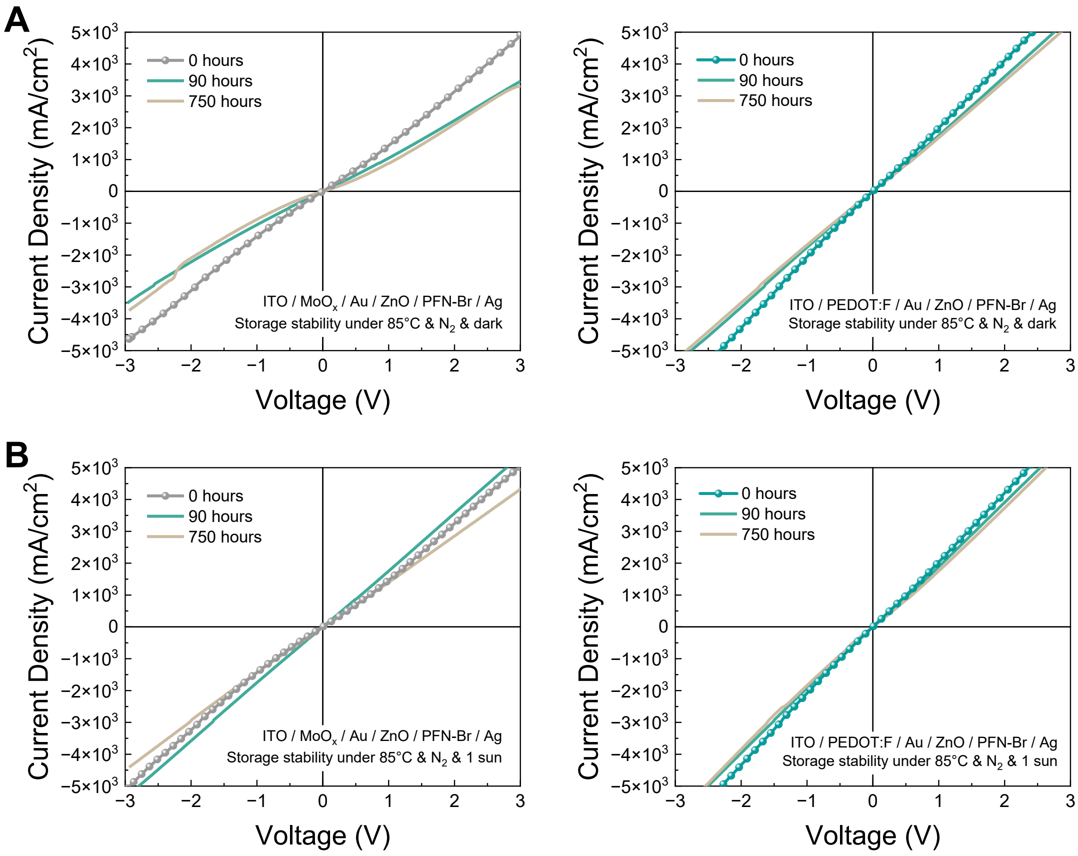

Figure S38. Storage stability of ITO/MoO_x or PEDOT:F/Au/ZnO/PFN-Br devices under (A) 85°C & N_2 & dark condition, and (B) 85°C & N_2 & 1 sun condition.

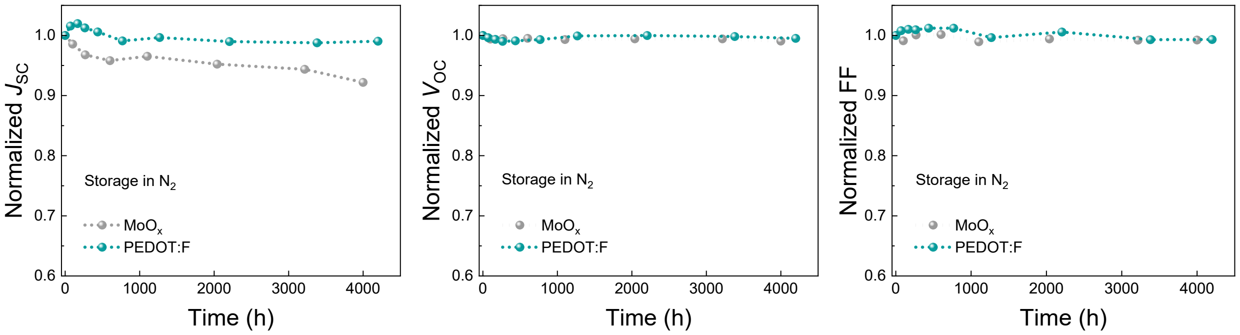

Figure S39. Normalized device parameters from *J-V* curves in Figures 5A and 5B.

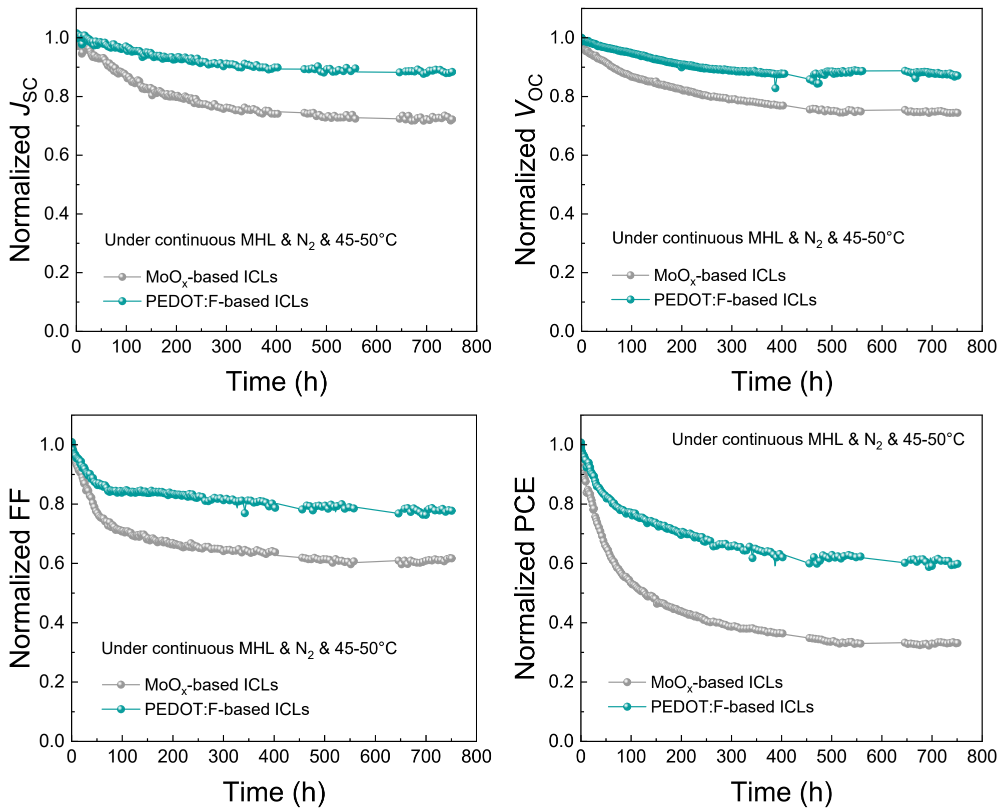

Figure S40. Normalized long-term operational stability of $CsPbI_2Br$-D18-Cl:L8-BO P-O-TSCs employing MoO_x and PEDOT:F at the short-circuit mode, under continuous metal-halide lamp (MHL) illumination with an intensity of 85 mW/cm^2 in N_2 at 45-50°C.

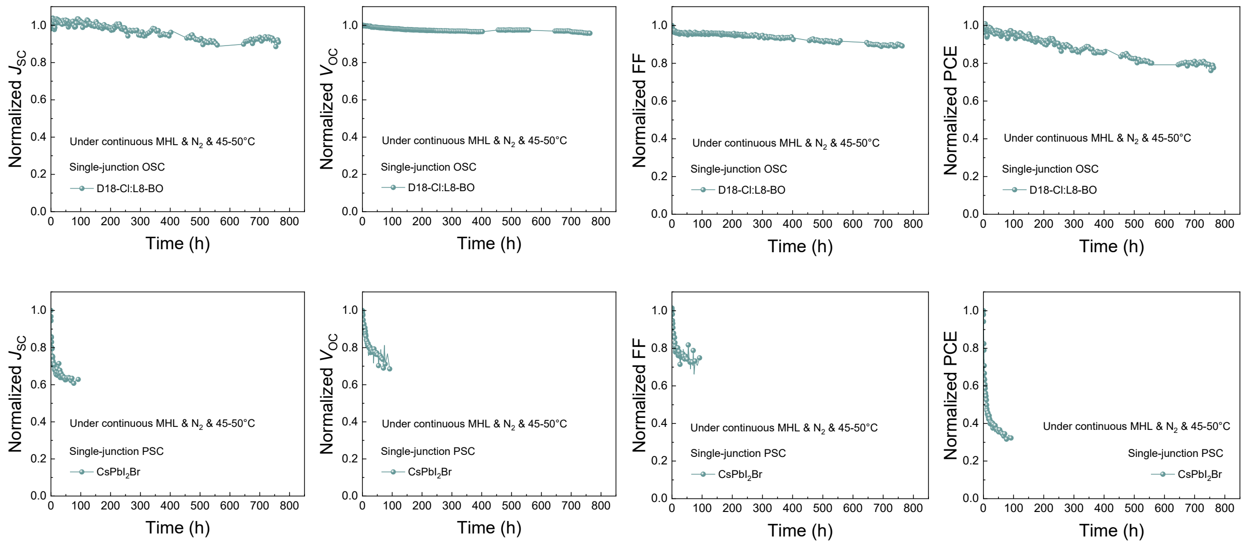

Figure S41. Normalized long-term operational stability of single-junction D18-Cl:L8-BO OSC and $CsPbI_2Br$ PSC at the short-circuit mode, under continuous metal-halide lamp (MHL) illumination with an intensity of 85 mW/cm^2 in N_2 at 45-50°C.

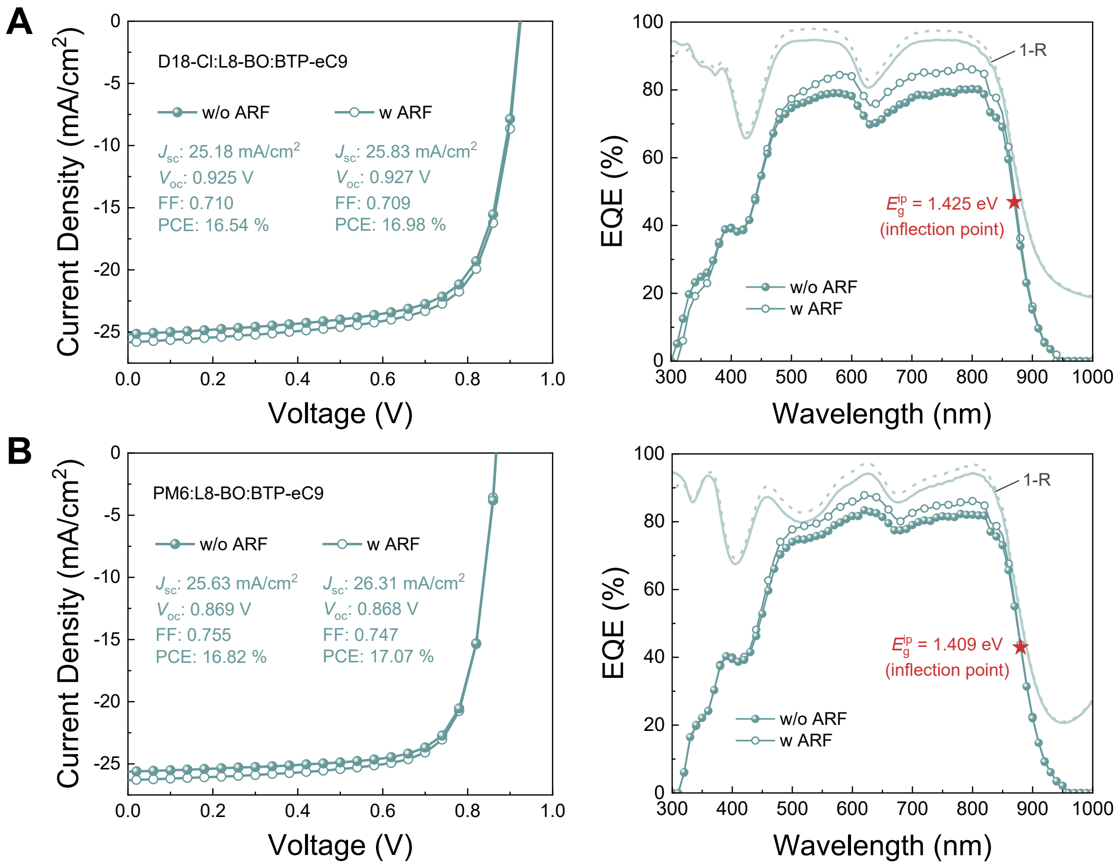

Figure S42. Device performance and corresponding EQE spectra of single-junction (A) D18-Cl:L8-BO:BTP-eC9 and (B) PM6:L8-BO:BTP-eC9 OSCs with or without an anti-reflection film (ARF). Note that the device structure is ITO/ZnO/PFN-Br/BHJ/2PACz-Cl/MoO_x/Ag. Reflection (denoted as 1-R) curves of the devices are also presented.

Table S8. Device parameters of $CsPbI_2Br$-D18-Cl:L8-BO:BTP-eC9 TSCs with MoO_x-based ICLs and PEDOT:F-based ICLs, and the corresponding sub-cells.

Device		V_{OC} (V)	J_{SC} (mA/cm^2)	FF (%)	PCE (%)
TSC (w ARF)	MoO_x-based ICLs	2.25	11.95	72.66	19.58
	PEDOT:F-based ICLs	2.29	14.69	72.52	24.39
Front (w/o ARF)		1.40	15.83	78.46	17.44
Rear (w/o ARF)		0.925	25.18	71.00	16.54

Table S9. Device performance of champion CsPbI2Br-D18-Cl:L8-BO:BTP-eC9 TSC with the PEDOT:F-based ICLs under reverse and forward scans.

Scan Direction	V_{OC} (V)	J_{SC} (mA/cm^2)	FF (%)	PCE (%)
Reverse	2.29	14.69	72.52	24.39
Forward	2.24	14.70	65.56	21.65

Table S10. Device parameters of CsPbI2Br-PM6:L8-BO:BTP-eC9 TSCs with MoO_x-based ICLs and PEDOT:F-based ICLs, and the corresponding sub-cells.

Device		V_{OC} (V)	J_{SC} (mA/cm^2)	FF (%)	PCE (%)
TSC (w ARF)	MoO_x-based ICLs	2.21	13.41	77.79	23.15
	PEDOT:F-based ICLs	2.23	14.98	75.75	25.34
Front (w/o ARF)		1.40	15.83	78.46	17.44
Rear (w/o ARF)		0.869	25.63	75.50	16.82

Table S11. Device performance of champion CsPbI2Br-PM6:L8-BO:BTP-eC9 TSC with the PEDOT:F-based ICLs under reverse and forward scans.

Scan Direction	V_{OC} (V)	J_{SC} (mA/cm^2)	FF (%)	PCE (%)
Reverse	2.23	14.98	75.75	25.34
Forward	2.18	14.97	73.40	23.88

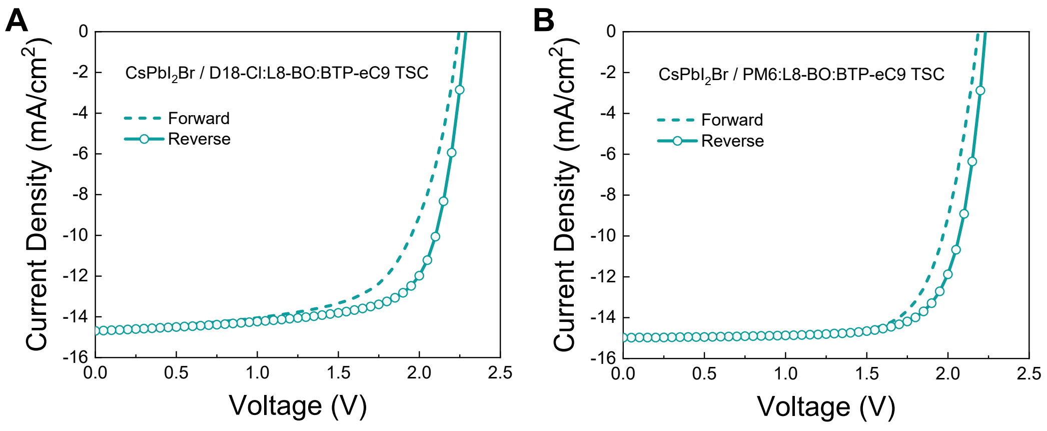

Figure S43. *J-V* curves of (A) champion CsPbI2Br-D18-Cl:L8-BO:BTP-eC9 TSC and (B) champion CsPbI2Br-PM6:L8-BO:BTP-eC9 TSC under reverse and forward scans.

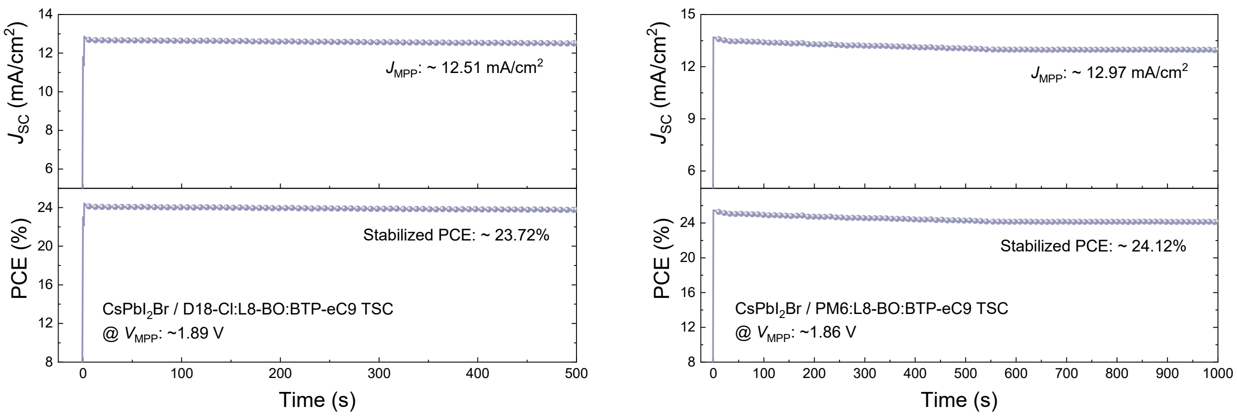

Figure S44. Corresponding maximum power point (MPP) tracking of champion $CsPbI_2Br$/D18-Cl:L8-BO:BTP-eC9 and $CsPbI_2Br$/PM6:L8-BO:BTP-eC9 TSCs under solar simulator in air.

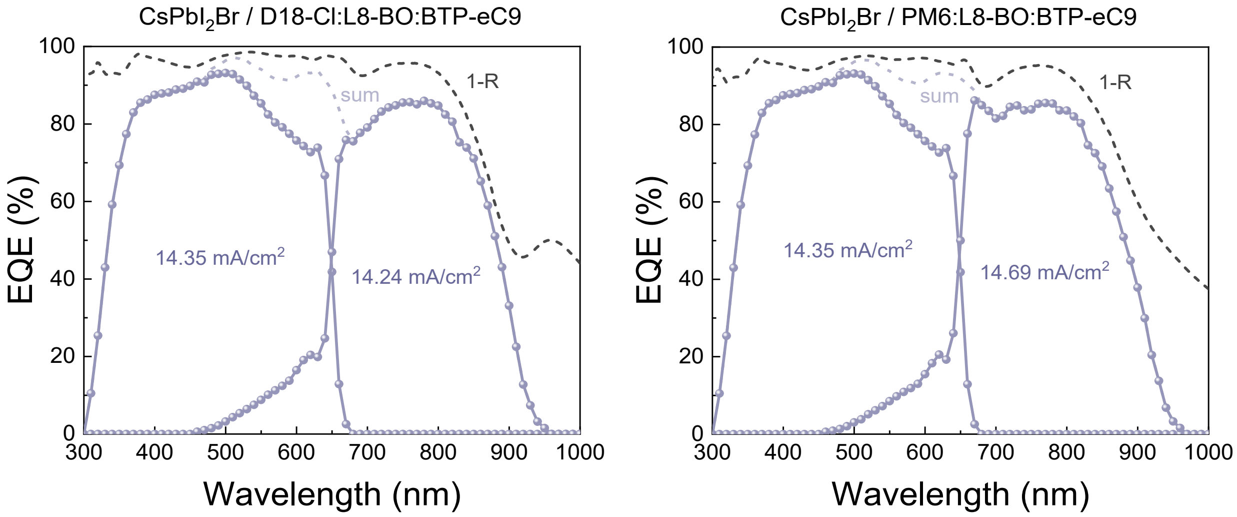

Figure S45. Corresponding EQE spectra of the champion P-O-TSCs utilizing PEDOT:F, incorporating D18-Cl:L8-BO:BTP-eC9 and PM6:L8-BO:BTP-eC9 organic rear cells. Reflection (denoted as 1-R) and sum (total EQE of individual sub-cells) curves are also presented.

Supplementary References

1 Li, D. & Neumann, A. W. Contact angles on hydrophobic solid surfaces and their interpretation. *J. Colloid Interface Sci.* **148**, 190-200 (1992).

2 Kozbial, A. *et al.* Study on the surface energy of graphene by contact angle measurements. *Langmuir* **30**, 8598-8606 (2014).

3 Wenzel, R. N. RESISTANCE OF SOLID SURFACES TO WETTING BY WATER. *Industrial & Engineering Chemistry* **28**, 988-994 (1936).

4 Chen, X. *et al.* Efficient and Reproducible Monolithic Perovskite/Organic Tandem Solar Cells with Low-Loss Interconnecting Layers. *Joule* **4**, 1594-1606 (2020).

5 Xie, Y. M. *et al.* Homogeneous Grain Boundary Passivation in Wide-Bandgap Perovskite Films Enables Fabrication of Monolithic Perovskite/Organic Tandem Solar Cells with over 21% Efficiency. *Adv. Funct. Mater.* **32**, 2112126 (2022).

6 Chen, W. *et al.* Monolithic perovskite/organic tandem solar cells with 23.6% efficiency enabled by reduced voltage losses and optimized interconnecting layer. *Nat. Energy* **7**, 229-237 (2022).

7 Brinkmann, K. O. *et al.* Perovskite-organic tandem solar cells with indium oxide interconnect. *Nature* **604**, 280-286 (2022).

8 Qin, S. *et al.* Constructing Monolithic Perovskite/Organic Tandem Solar Cell with Efficiency of 22% via Reduced Open-Circuit Voltage Loss and Broadened Absorption Spectra. *Adv. Mater.* **34**, e2108829 (2022).

9 Zhang, Z. *et al.* High-Performance 1 cm^2 Perovskite-Organic Tandem Solar Cells with a Solvent-Resistant and Thickness-Insensitive Interconnecting Layer. *ACS Appl. Mater. Interfaces* **14**, 29896-29904 (2022).

10 Xie, Y.-M. *et al.* Understanding the role of interconnecting layer on determining monolithic perovskite/organic tandem device carrier recombination properties. *J. Energy Chem.* **71**, 12-19 (2022).

11 Xu, H. *et al.* Metal-Free Interconnecting Layer for Monolithic Perovskite/Organic Tandem Solar Cells with Enhanced Outdoor Stability. *ACS Appl. Energy Mater.* **5**, 14035-14044 (2022).

12 Wu, S. *et al.* Redox mediator-stabilized wide-bandgap perovskites for monolithic perovskite-organic tandem solar cells. *Nat. Energy* **9**, 411-421 (2024).

13 Zhang, Z. *et al.* Suppression of phase segregation in wide-bandgap perovskites with thiocyanate ions for perovskite/organic tandems with 25.06% efficiency. *Nat. Energy* **9**, 592-601 (2024).

14 Guo, X. *et al.* Stabilizing efficient wide-bandgap perovskite in perovskite-organic tandem solar cells. *Joule*, doi:10.1016/j.joule.2024.06.009 (2024).

15 Aqoma, H. *et al.* High-Efficiency Solution-Processed Two-Terminal Hybrid Tandem Solar Cells Using Spectrally Matched Inorganic and Organic Photoactive Materials. *Adv. Energy Mater.* **10**, 2001188 (2020).

16 Xie, S. *et al.* Efficient monolithic perovskite/organic tandem solar cells and their efficiency potential. *Nano Energy* **78**, 105238 (2020).

17 Wang, P. *et al.* Tuning of the Interconnecting Layer for Monolithic Perovskite/Organic Tandem Solar Cells with Record Efficiency Exceeding 21%. *Nano Lett.* **21**, 7845-7854 (2021).

18 Chen, W. *et al.* Surface Reconstruction for Stable Monolithic All-Inorganic Perovskite/Organic Tandem Solar Cells with over 21% Efficiency. *Adv. Funct. Mater.* **32**, 2109321 (2021).

19 Liu, L., Xiao, Z., Zuo, C. & Ding, L. Inorganic perovskite/organic tandem solar cells with efficiency over 20%. *Journal of Semiconductors* **42**, 020501 (2021).

20 Gu, X. *et al.* Organic Solar Cell With Efficiency Over 20% and V_{OC} Exceeding 2.1 V Enabled by Tandem With All-Inorganic Perovskite and Thermal Annealing-Free Process. *Adv. Sci.* **9**, e2200445 (2022).

21 Sun, S. Q. *et al.* All-Inorganic Perovskite-Based Monolithic Perovskite/Organic Tandem Solar Cells with 23.21% Efficiency by Dual-Interface Engineering. *Adv. Energy Mater.* **13**, 2204347 (2023).

22 Yang, H. *et al.* Regulating Charge Carrier Recombination in the Interconnecting Layer to Boost the Efficiency and Stability of Monolithic Perovskite/Organic Tandem Solar Cells. *Adv. Mater.* **35**, e2208604 (2023).

23 Jiang, S. *et al.* Synergistic electrical and light management enables efficient monolithic inorganic perovskite/organic tandem solar cells with over 24% efficiency. *Energy & Environ. Sci.* **17**, 219-226 (2024).

24 Zhou, Q. *et al.* Tailored Lattice "Tape" to Confine Tensile Interface for 11.08%-Efficiency All-Inorganic $CsPbBr_3$ Perovskite Solar Cell with an Ultrahigh Voltage of 1.702 V. *Adv. Sci.* **8**, e2101418 (2021).

25 Zhang, J. *et al.* A Universal Grain "Cage" to Suppress Halide Segregation of Mixed-Halide Inorganic Perovskite Solar Cells. *ACS Energy Lett.* **7**, 3467-3475 (2022).

26 Zhang, Y. *et al.* High Performance Thick-Film Nonfullerene Organic Solar Cells with Efficiency over 10% and Active Layer Thickness of 600 nm. *Adv. Energy Mater.* **9**, 1902688 (2019).

27 Tian, J. *et al.* Quantifying the Energy Losses in $CsPbI_2Br$ Perovskite Solar Cells with an Open-Circuit Voltage of up to 1.45 V. *ACS Energy Lett.* **7**, 4071-4080 (2022).

28 Ye, Q. *et al.* Stabilizing gamma-$CsPbI_3$ Perovskite via Phenylethylammonium for Efficient Solar Cells with Open-Circuit Voltage over 1.3 V. *Small* **16**, e2005246 (2020).

29 Chen, Y. *et al.* Dual Passivation of Perovskite and SnO_2 for High-Efficiency $MAPbI_3$ Perovskite Solar Cells. *Adv. Sci.* **8**, 2001466 (2021).

30 Min, H. *et al.* Perovskite solar cells with atomically coherent interlayers on SnO_2 electrodes. *Nature* **598**, 444-450 (2021).
