# Supplementary material for: Overcoming optical losses in thin metal-based recombination layers for efficient n-i-p perovskite-organic tandem solar cells
Source: Nat Commun. 2025 Jan 2;16:154. doi: 10.1038/s41467-024-55376-7 (PMC11696673; doi:10.1038/s41467-024-55376-7)
Supplement: Supplementary file 3 — Reporting Summary [file 41467_2024_55376_MOESM3_ESM.pdf]

nature portfolio

Corresponding author(s): Chao Liu, Larry Lüer, Christoph J. Brabec

Solar Cells Reporting Summary

Nature Portfolio wishes to improve the reproducibility of the work that we publish. This form is intended for publication with all accepted papers reporting the characterization of photovoltaic devices and provides structure for consistency and transparency in reporting. Some list items might not apply to an individual manuscript, but all fields must be completed for clarity.

For further information on Nature Research policies, including our data availability policy, see Authors & Referees.

Experimental design

Please check the following details are reported in the manuscript, and provide a brief description or explanation where applicable.

1. Dimensions

Area of the tested solar cells

☒ Yes — Active area: 0.038 cm2. Described in 'Methods' section
☐ No — *Explain why this information is not reported/not relevant.*

Method used to determine the device area

☒ Yes — All the device areas are determined by shadow masks
☐ No — *Explain why this information is not reported/not relevant.*

2. Current-voltage characterization

Current density-voltage (J-V) plots in both forward and backward direction

☒ Yes — Supplementary Figures S22, S26, and S43
☐ No

Voltage scan conditions

☒ Yes — The J-V characteristics were performed at a scan rate of 20 ms/step and a scan step of 50 mV, as described in Methods, section 'Film and device characterization'.
☐ No — *Explain why this information is not reported/not relevant.*

Test environment

☒ Yes — Current density-voltage characteristics (J-V) were characterized under ambient condition at room temperature as described in Methods, section 'Film and device characterization'.
☐ No — *Explain why this information is not reported/not relevant.*

Protocol for preconditioning of the device before its characterization

☒ Yes — During the measurement, an anti-reflection film was applied to all tandem devices as described in Methods, section 'Film and device characterization'.
☐ No — *Explain why this information is not reported/not relevant.*

Stability of the J-V characteristic

☒ Yes — Figure S5, Supplementary Figures S27, S39, S40, S41, and S44
☐ No — *Explain why this information is not reported/not relevant.*

3. Hysteresis or any other unusual behaviour

Description of the unusual behaviour observed during the characterization

☒ Yes — A small J-V hysteresis was observed in tandem devices as shown in Figures S26 and S43. There was no J-V hysteresis for single-junction organic solar cells (D18-Cl:L8-BO) in Figure S22B. Single-junction perovskite solar cells showed the hysteresis in Figure S22A. There is no other unusual behavior during the characterization.
☐ No — *Explain why this information is not reported/not relevant.*

Related experimental data

☒ Yes — They are provided in Supplementary Figures S22, S26 and S43
☐ No — *Explain why this information is not reported/not relevant.*

4. Efficiency

External quantum efficiency (EQE) or incident photons to current efficiency (IPCE)

☒ Yes — EQE spectra of solar cells were recorded on a commercial EQE measurement system (Taiwan, Enlitech, QE-R) under ambient condition and the light intensity at each wavelength was calibrated with a standard single-crystal Si photovoltaic cell. Described in Methods, section 'Film and device characterization'.
☐ No — *Explain why this information is not reported/not relevant.*

A comparison between the integrated response under the standard reference spectrum and the response measure under the simulator

☒ Yes ☐ No

Figures 4B and 4C; Figures 4G, 4H and S45.

Explain why this information is not reported/not relevant.

For tandem solar cells, the bias illumination and bias voltage used for each subcell

☒ Yes ☐ No

For EQE measurement of tandem cells, the perovskite front sub-cells were measured while saturating the organic rear sub-cells with continuous light from an 808 nm laser (LDM808/3LJ), while the organic rear sub-cells were measured while saturating the perovskite front sub-cells with continuous light from a 450 nm laser (CW450-05). No bias voltage was applied for the EQE measurement of both sub-cells. During the measurement, an anti-reflection film was applied to all devices. Described in Methods, section 'Film and device characterization'.

Explain why this information is not reported/not relevant.

5. Calibration

Light source and reference cell or sensor used for the characterization

☒ Yes ☐ No

Current density-voltage characteristics (J-V) were measured with a Keithley 2400. The illumination was provided by a WAVELABS SINUS-70 3A solar simulator with AM1.5G spectra at 100 mW cm-2 under ambient condition. The light intensity was calibrated with a crystalline Si cell.

Explain why this information is not reported/not relevant.

Confirmation that the reference cell was calibrated and certified

☒ Yes ☐ No

The light intensity was calibrated with a standard Si reference cell (91150V) bought from Newport.

Explain why this information is not reported/not relevant.

Calculation of spectral mismatch between the reference cell and the devices under test

☒ Yes ☐ No

A standard silicon reference cell was used as the reference for the EQE and JV measurements

Explain why this information is not reported/not relevant.

6. Mask/aperture

Size of the mask/aperture used during testing

☒ Yes ☐ No

0.038 cm2

Explain why this information is not reported/not relevant.

Variation of the measured short-circuit current density with the mask/aperture area

☐ Yes ☒ No

Report the difference in the short-circuit current density values measured with the mask and aperture area.

There is no variation of the measured short-circuit current densities with different mask/aperture area for small-area devices.

7. Performance certification

Identity of the independent certification laboratory that confirmed the photovoltaic performance

☐ Yes ☒ No

Identify the independent certification laboratory.

We focused on the optimization of the current tandem architecture to overcome the existing limitations, and we did not aim to claim a world-record efficiency.

A copy of any certificate(s)

☐ Yes ☒ No

Certificate copies should be provided in the Supplementary information. Please state the supplementary item number.

We focused on the optimization of the current tandem architecture to overcome the existing limitations, and we did not aim to claim a world-record efficiency.

8. Statistics

Number of solar cells tested

☒ Yes ☐ No

For CsPbI2Br/D18-Cl:L8-BO tandem, 25 devices were fabricated and measured (Figures 4E and 4F). For CsPbI2Br/D18-Cl:L8-BO:BTP-eC9 and CsPbI2Br/PM6:L8-BO:BTP-eC9 tandems, 20 devices were fabricated and measured (Figure 4I)

Explain why this information is not reported/not relevant.

Statistical analysis of the device performance

☒ Yes ☐ No

We provided statistical analysis in Figures 4E, 4F, 4I, S24 and S25.

Explain why this information is not reported/not relevant.

9. Long-term stability analysis

Type of analysis, bias conditions and environmental conditions

☒ Yes ☐ No

The long-term storage stability tests were performed on the unencapsulated solar cells in N2 atmosphere under dark and room temperature conditions (Figure 5 and Figure S39). The measurement was in the open air.
The long-term operational stability of the devices was assessed at the short-circuit mode, under continuous metal-halide lamp (MHL) illumination with an intensity of 85 mW/cm2 in a nitrogen-filled chamber, with the temperature maintained at 45-50°C (Figures S40-S41). The MHL provided light in the wavelength range of 400 to 800 nm.

Explain why this information is not reported/not relevant.
